# ADHD-anchored partitioning of non-small cell lung cancer genetic covariance identifies *CLPTM1L* and suggestive innate-immune signals

**DOI:** 10.3389/fimmu.2026.1913958

**Published:** 2026-08-13

**Authors:** Ting Yu, Peng Wang, Ziwei Zheng, Kun Zhao, Gang Yan, Jiawen Song, Xinglin Yi, Shaoling Yang, Zhibiao Jiang

**Affiliations:** 1 Department of Radiology, Shanghai Jiaotong University Affiliated Sixth People’s Hospital South Campus, Shanghai, China; 2 Department of General Practice, Nanqiao Town Community Health Service Center, Fengxian, Shanghai, China; 3 Department of Ultrasonography, Shanghai Eighth People’s Hospital, Shanghai, China; 4 Department of Thoracic Surgery, Shanghai Jiaotong University Affiliated Sixth People’s Hospital South Campus, Shanghai, China; 5 Department of Respiratory Center, Nanjing University, Nanjing, China

**Keywords:** ADHD, C-reactive protein, genomic structural equation modelling, innate immunity, non-small cell lung cancer

## Abstract

**Background:**

Individuals with attention-deficit/hyperactivity disorder (ADHD) show an elevated incidence of non-small cell lung cancer (NSCLC), usually attributed to smoking. Innate immune activation and chronic inflammation are implicated in both, yet whether shared germline genetics include a component statistically independent of the measured current-smoking phenotype, and whether that liability has an innate immune dimension, remains unclear.

**Methods:**

We applied genomic structural equation modelling to GWAS summary statistics to partition genetic covariance into indirect and conditional direct components with respect to measured current smoking. Among twelve psychiatric and personality traits, only ADHD survived FDR and served as the primary exposure; neuroticism was retained as a secondary exploratory indicator. Component statistics underwent gene-level and tissue-enrichment analyses. We estimated ADHD and NSCLC genetic correlations with inflammatory biomarkers, tested the NSCLC signal for innate-pathway enrichment, and examined *CLPTM1L* response to non-nicotine stress in lung adenocarcinoma cells.

**Results:**

We found that *CLPTM1L*, *IREB2* and the chromosome 15q25 nicotinic-receptor cluster were prioritised within a component statistically independent of the measured current-smoking phenotype, corresponding to approximately 38% of the model-based shared genetic covariance, with lung and brain enrichment. Pathway and biomarker analyses suggested an innate immune and inflammatory dimension, but did not establish a mechanism. ADHD and NSCLC were each positively genetically correlated with C-reactive protein (ADHD rg=0.26, *P=*8.2×10^-^¹³; NSCLC rg=0.13, *P* = 0.0074); additional inflammatory-trait comparisons were exploratory. The NSCLC signal showed nominal enrichment of innate pattern-recognition-receptor and NF-κB/antiviral pathways. In A549 cells, *CLPTM1L* protein increased under genotoxic and hypoxic stress, with immunofluorescence 3.4-fold and 2.3-fold above control.

**Conclusions:**

An approximate model-based component of the ADHD–NSCLC genetic covariance was statistically independent of the measured current-smoking phenotype; pathway and biomarker analyses suggested, but did not establish, an innate immune and inflammatory dimension alongside the 15q25 cholinergic axis. *CLPTM1L* expression was responsive in a non-nicotine pathological cell state, without evidence of mediation or causality. These hypothesis-generating findings nominate innate-immune hypotheses for functional follow-up.

## 1 Introduction

Psychiatric morbidity is accompanied by higher rates of physical disease and premature mortality (1). An elevated incidence of lung cancer has been documented in people with psychiatric conditions, including schizophrenia, depression and ADHD (2). A much higher prevalence of cigarette smoking is the major recognised contributor, yet it does not fully explain the association (3).

Statistical-genetic work indicates that this co-occurrence is partly rooted in shared genetic architecture rather than behaviour alone (4, 5). Genetic correlations have been reported between psychiatric traits and lung cancer, with ADHD liability showing the clearest evidence in relation to lung-cancer risk (6). ADHD has genetic links to smoking-related behaviours, whereas the neuroticism literature provides context for exploratory inclusion rather than establishing a smoking association (7, 8). These observations motivate testing whether the ADHD–NSCLC genetic covariance contains a model-defined component associated with measured current smoking and a residual component not captured by that phenotype; they do not establish separate biological routes.

Disentangling such multivariate relationships requires methods that move beyond pairwise correlation. Genomic structural equation modelling represents the joint genetic architecture of several traits and can decompose conditional genetic covariance into a model-defined component associated with measured current smoking and a conditional component not captured by that phenotype (9). Its value here does not depend on a latent factor. The framework decomposes the genetic covariance among an exposure, the smoking mediator and NSCLC, and it generates component-specific summary statistics for downstream biological analysis.

We therefore used this framework to dissect the genetic covariance linking an ADHD-anchored liability to NSCLC. Rather than assuming a broad psychopathology construct, we screened twelve psychiatric and personality traits and let the data define the exposure. ADHD was the only trait to survive correction and was the primary exposure; neuroticism was retained only as a secondary exploratory indicator. Our primary aim was to quantify the model-defined components associated with, and not captured by, the measured current-smoking phenotype, and to identify the loci, genes and tissues underlying each component. We then asked, in a minimal cell experiment, whether the prioritised candidate gene *CLPTM1L* is responsive to non-nicotine pathological stress.

## 2 Methods

### 2.1 GWAS summary statistics and trait screening

To define the traits with shared genetic architecture, we assembled publicly available summary statistics for NSCLC, current smoking and twelve psychiatric and personality traits. NSCLC data were from the FinnGen consortium release 12 (10) and smoking data were from the MRC-IEU consortium. The twelve psychiatric and personality traits were obtained from the Psychiatric Genomics Consortium, GWAS Catalog and allied resources. Dataset accessions, sample sizes, ancestry, genome builds and provider links are provided in **Supplementary Table 1**. The core NSCLC and ADHD datasets were predominantly European-ancestry (NSCLC N = 385,195; ADHD 38,691 cases and 186,843 controls). Public summary statistics contain no participant-level identifiers, so sample overlap could not be assessed directly.

### 2.2 Genetic correlation and exposure selection

**Figure 1:**
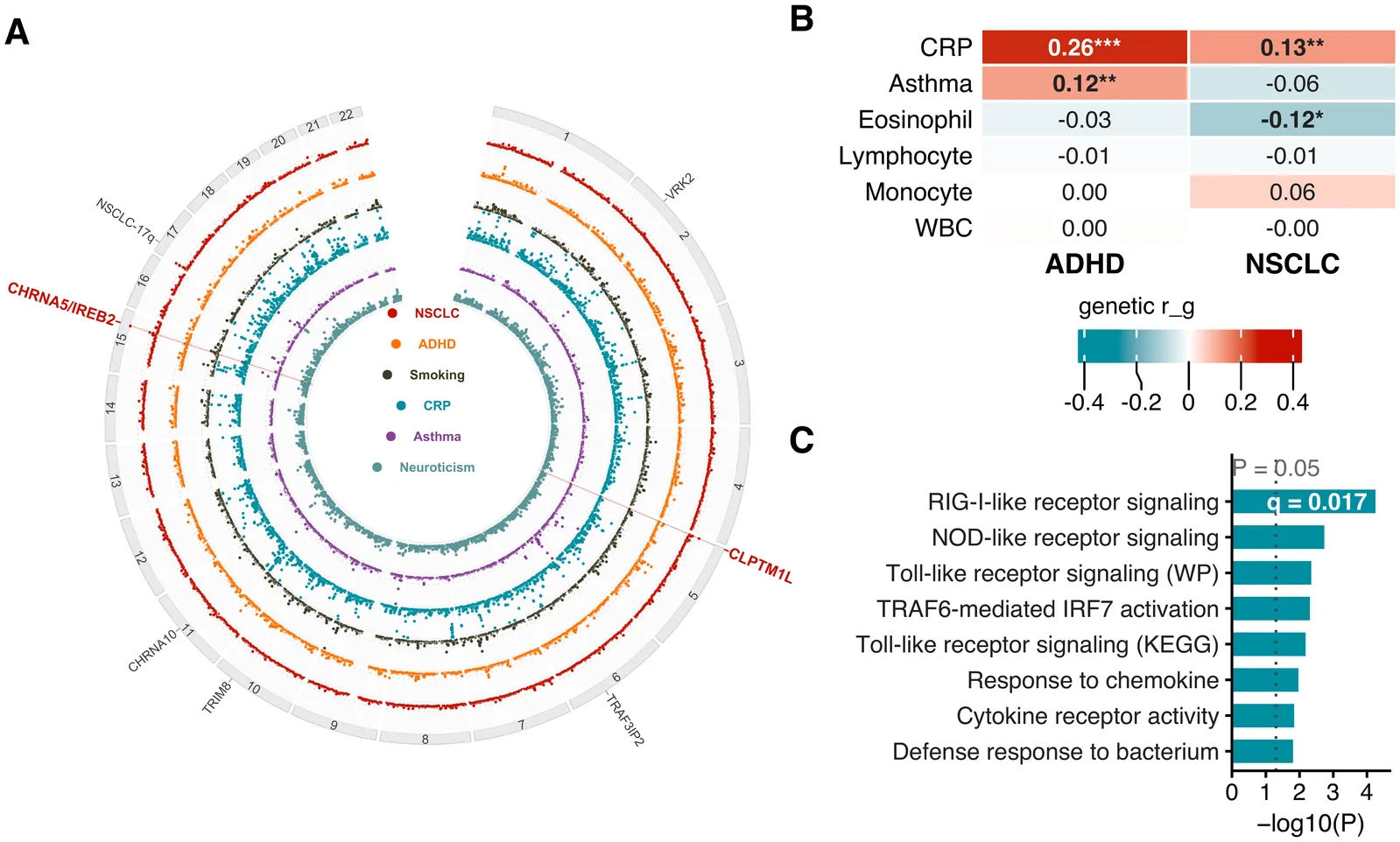
Suggestive innate immune and inflammatory signals associated with ADHD–NSCLC genetic covariance. **(A)** Circular genome-wide association plot showing, from outer to inner track, the −log10(P) signal for NSCLC, ADHD, smoking, C-reactive protein, asthma and neuroticism; loci assigned to the direct component (15q25 nicotinic-receptor cluster including *CHRNA5* and *IREB2*; 5p15.33/*CLPTM1L*) and separately annotated candidate genes (*TRAF3IP2*, *TRIM8*, *VRK2*) are labelled. **(B)** Bivariate LD-score-regression genetic correlations (rg) of ADHD and NSCLC with C-reactive protein, asthma and circulating eosinophil, lymphocyte, monocyte and white-blood-cell counts; *P < 0.05, **P < 0.01, ***P < 0.001. **(C)** MAGMA competitive gene-set enrichment of the NSCLC gene-level signal for innate immune pathways, shown as −log10(P); the dotted line marks nominal *P* = 0.05. RIG-I-like receptor signalling ranked fifth among 16,785 gene sets (*P* = 5.6×10^-5^). The q=0.017 value applies only to the innate-pathway subset. The innate enrichments did not survive correction across all 16,785 gene sets and are interpreted as suggestive.

To select a robust exposure, we estimated the genetic correlation between NSCLC and each trait by bivariate linkage-disequilibrium score regression (LDSC) with European LD-score reference files (11). P-values were corrected across the eleven interpretable traits by the Benjamini-Hochberg false-discovery rate. Panic disorder (ANX) yielded an out-of-bounds observed-scale heritability estimate (h²_obs=−0.022, SE = 0.079) and was therefore excluded before FDR correction. The negative correlations for anorexia nervosa, obsessive-compulsive disorder and Tourette syndrome were retained descriptively but were not used as positive indicators of the ADHD-anchored model. ADHD was the only trait whose correlation with NSCLC survived a 5% false-discovery rate (rg=0.24, q=2.6 × 10^-4^) and was used as the primary exposure. Neuroticism showed a nominal positive correlation that did not survive correction (rg=0.13, q=0.056) and was retained only as a secondary exploratory indicator, with neuroticism-based results treated as exploratory. The signs of these correlations were retained for transparent reporting and were not interpreted mechanistically. Full results with signs and q-values are reported in **Supplementary Table 2**. LDSC was run with version 2.0.0 and the European LD-score reference. The primary NSCLC and ADHD LDSC diagnostics were h²_obs=0.0061 (SE = 0.0015; intercept=1.0532, SE = 0.0087) and h²_obs=0.0384 (SE = 0.0020; intercept=1.0717, SE = 0.0105), respectively; the cross-trait intercept was 0.0085 (SE = 0.0063). These diagnostic values are reported to provide context for the correlation estimates. Coordinate-based annotation used source/tool-specific builds (including NCBI38 for MAGMA and hg19 for **Figure 1** plotting).

### 2.3 Genomic structural equation modelling

To partition the shared genetic effect, we fitted, in genomic structural equation modelling (Genomic SEM), a mediation model in which an ADHD-anchored liability, with ADHD as the primary indicator (loading fixed to 1) and neuroticism as a secondary exploratory indicator, was related to the measured current-smoking phenotype and NSCLC. Path a was the liability-to-smoking path, path b the smoking-to-NSCLC path, the indirect component was a×b, and the direct c′ component was statistically independent of the measured smoking phenotype (9). Genomic SEM v0.0.5 was used. The path coefficients partition genetic covariance estimated from score regression and do not represent individual-level causal effects. The direct and indirect labels denote summary-statistics genetic-covariance components conditional on the measured current-smoking phenotype; they do not imply biological independence or individual-level causation. ADHD was the primary indicator (loading fixed to 1); neuroticism was retained only as a secondary exploratory indicator and does not define a general psychopathology or p-factor construct. Component-specific summary statistics were generated for downstream annotation: the direct component is a conditional NSCLC statistic after accounting for measured current smoking, and the indirect component is derived from the smoking-mediator component. The resulting percentages are interpreted as approximate model-based partitions of genetic covariance.

### 2.4 Post-GWAS annotation, gene-based tests and enrichment

To move from variants to genes and tissues, we annotated the component-specific statistics, defined genomic risk loci and lead variants, mapped variants to genes and scored variant deleteriousness using Functional Mapping and Annotation of Genome-wide Association Studies (FUMA) (12). To obtain gene-level evidence, we performed gene-based association testing with MAGMA v1.10 using the SNP-wise mean model and gene-size, gene-density and inverse-MAC covariates (13) and prioritised candidate genes using the Fine-mapping Of CaUsal gene Sets (FOCUS) expression-based fine-mapping framework (14). To place the signals biologically, we tested tissue enrichment across 54 reference tissues from GTEx (15) and competitive pathway enrichment against curated gene sets (16–18). Candidate prioritisation was not interpreted as proof of causality, and multiple-testing correction was applied at each reported stage. MAGMA used the European 1000 Genomes reference, NCBI38 gene locations, a 35-kb upstream/10-kb downstream annotation window and SNP-wise mean gene scores.

### 2.5 Cell-based stress-response assay

To test whether the prioritised candidate gene *CLPTM1L* responds to non-nicotine pathological stress, we examined its expression in A549 human lung adenocarcinoma cells, which were authenticated by short-tandem-repeat profiling and confirmed mycoplasma-negative. Cells were assigned to three conditions, each in three independent biological replicates. A vehicle condition served as control, a sub-lethal genotoxic condition used cisplatin for 24 h, and a hypoxic condition used 150 µM cobalt chloride for 24 h. The cisplatin dose was chosen by CCK-8 viability titration as the highest concentration retaining at least 70% viability, which corresponded to 6 µM. Protein expression was measured by Western blot for *CLPTM1L*, for the genotoxic-state markers γH2AX and CDKN1A, for the hypoxia-state markers CA9 and VEGFA, and for β-actin as loading control. Subcellular localisation was assessed by immunofluorescence for *CLPTM1L* and γH2AX with DAPI nuclear counterstain, and mean fluorescence intensity was quantified in ImageJ. The cell-state markers acted as a positive-control gate, and *CLPTM1L* was interpreted only when the relevant markers responded as expected. Group differences were tested by paired t test with significance at *P* < 0.05; pairing was defined by matched biological replicate/batch across conditions. Primary antibodies were anti-*CLPTM1L* (Abcam ab155119, rabbit polyclonal), anti-γH2AX (Phospho-Histone H2A.X Ser139, Sanying/Proteintech 29380-1-AP), anti-CDKN1A/p21 (Sanying/Proteintech 10355-1-AP), anti-CA9 (Sanying/Proteintech 11071-1-AP), anti-VEGFA (Sanying/Proteintech 19003-1-AP) and anti-β-actin (Sanying/Proteintech 66009-1-Ig).

### 2.6 Genetic correlation with inflammatory biomarkers

We estimated bivariate LDSC (11) genetic correlations of ADHD and NSCLC with C-reactive protein (19) and selected inflammatory traits (20), using European LD scores. These comparator analyses were exploratory; source accessions and sample metadata are provided in **Supplementary Table 1**.

### 2.7 Innate immune pathway enrichment

To localise the shared signal to biological programmes, we computed gene-level association statistics for NSCLC with MAGMA (13) under the SNP-wise mean model with a 1000 Genomes European reference (MAGMA v1.10; gene-size, gene-density and inverse-MAC covariates) and tested them for competitive gene-set enrichment against curated pathways from the Molecular Signatures Database (21) and KEGG (16), with attention to innate immune programmes (pattern-recognition-receptor, NF-κB, antiviral and cytokine signalling). Competitive tests were one-sided for positive enrichment. The confirmatory Bonferroni threshold was α=2.98×10^-6^ across 16,785 tested gene sets, and enrichments not passing that threshold were considered nominal. The RIG-I-like receptor result (*P* = 5.6×10^-5^) did not meet the global significance threshold. The q=0.017 value applies only to the selected innate-pathway subset. The NSCLC gene-level analysis here is distinct from the direct/indirect component statistics.

## 3 Results

### 3.1 ADHD shows a robust genetic correlation with NSCLC

Across the twelve-trait screen, ADHD was the only trait whose genetic correlation with NSCLC survived correction (rg=0.24, q=2.6×10^-4^), and ADHD was positively genetically correlated with smoking. Neuroticism showed only a nominal correlation (rg=0.13), anorexia nervosa, obsessive-compulsive disorder and Tourette syndrome were negatively correlated with NSCLC, and the remaining traits were null (**Supplementary Table 2**). ADHD therefore served as the primary exposure and neuroticism as a secondary exploratory indicator.

### 3.2 An indirect component associated with measured current smoking and a direct component statistically independent of measured smoking

**Figure 2:**
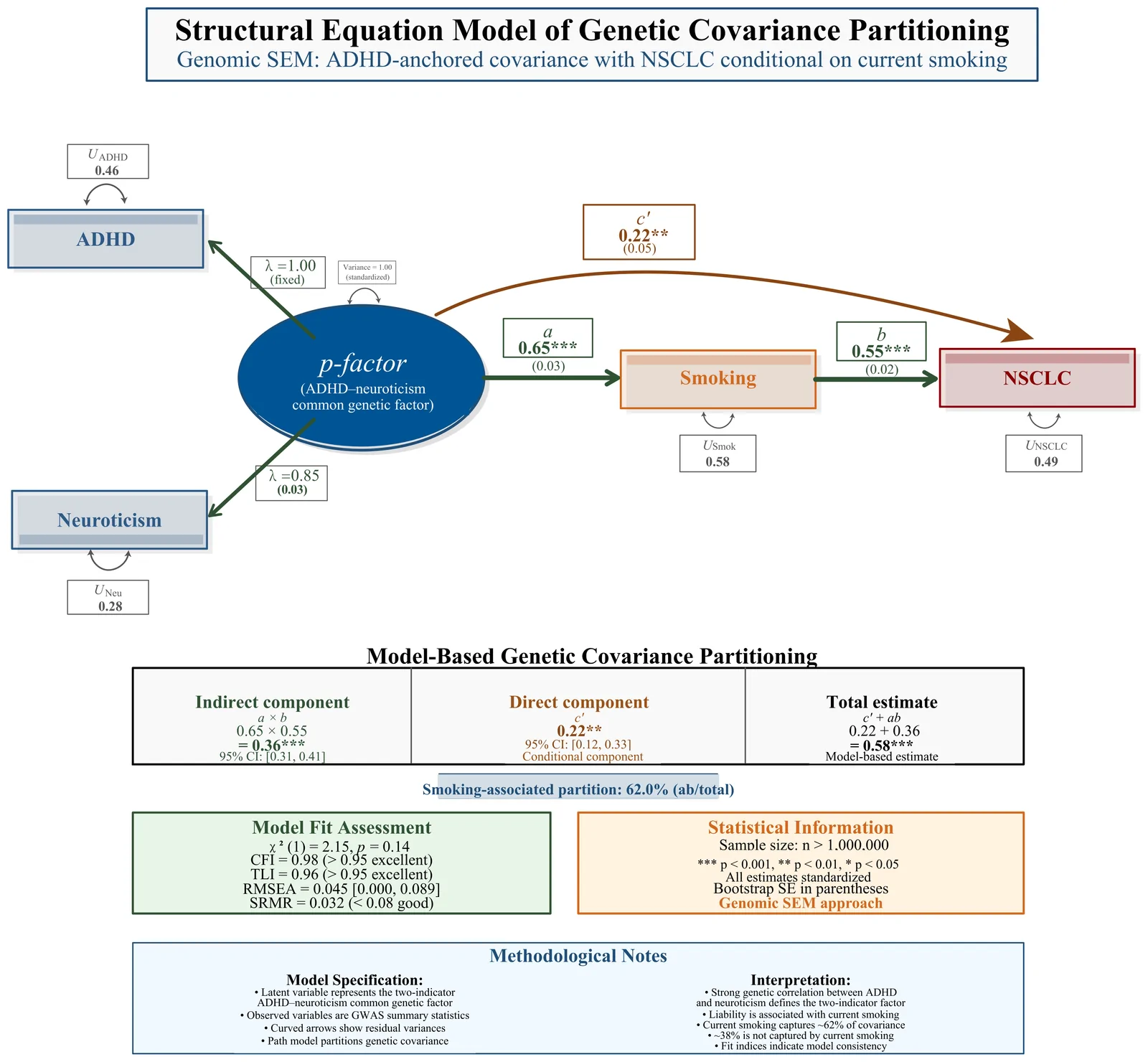
Genomic structural equation model partitioning ADHD-anchored genetic covariance with NSCLC conditional on measured current smoking. Path a links the liability to current smoking; path b links current smoking to NSCLC; path c-prime denotes the conditional direct component statistically independent of the measured current-smoking phenotype. The approximate 62%/38% values are model-based partitions of genetic covariance rather than biological mediation proportions. The liability is indexed primarily by ADHD; neuroticism is a secondary exploratory indicator and does not define a general psychopathology factor. In the figure, the “p-factor” label denotes only the two-indicator ADHD–neuroticism common genetic factor used in this model and is not presented as a validated general psychopathology factor.

The mediation model was statistically consistent with the data (comparative fit index 0.98, Tucker-Lewis index 0.96, standardised root-mean-square residual 0.032), where fit indices indicate consistency rather than confirmation of the true structure (**Figure 2**). The ADHD-anchored liability was positively associated with smoking, and smoking with NSCLC, and a conditional direct path to NSCLC remained after the smoking-associated component defined by measured current smoking was accounted for. Decomposition indicated that about 62% of the genetic covariance was associated with the measured current-smoking phenotype and about 38% was not captured by that phenotype; these values are interpreted as approximate model-based partitions rather than precise biological proportions.

### 3.3 Distinct loci and genes underlie the two components

**Figure 3:**
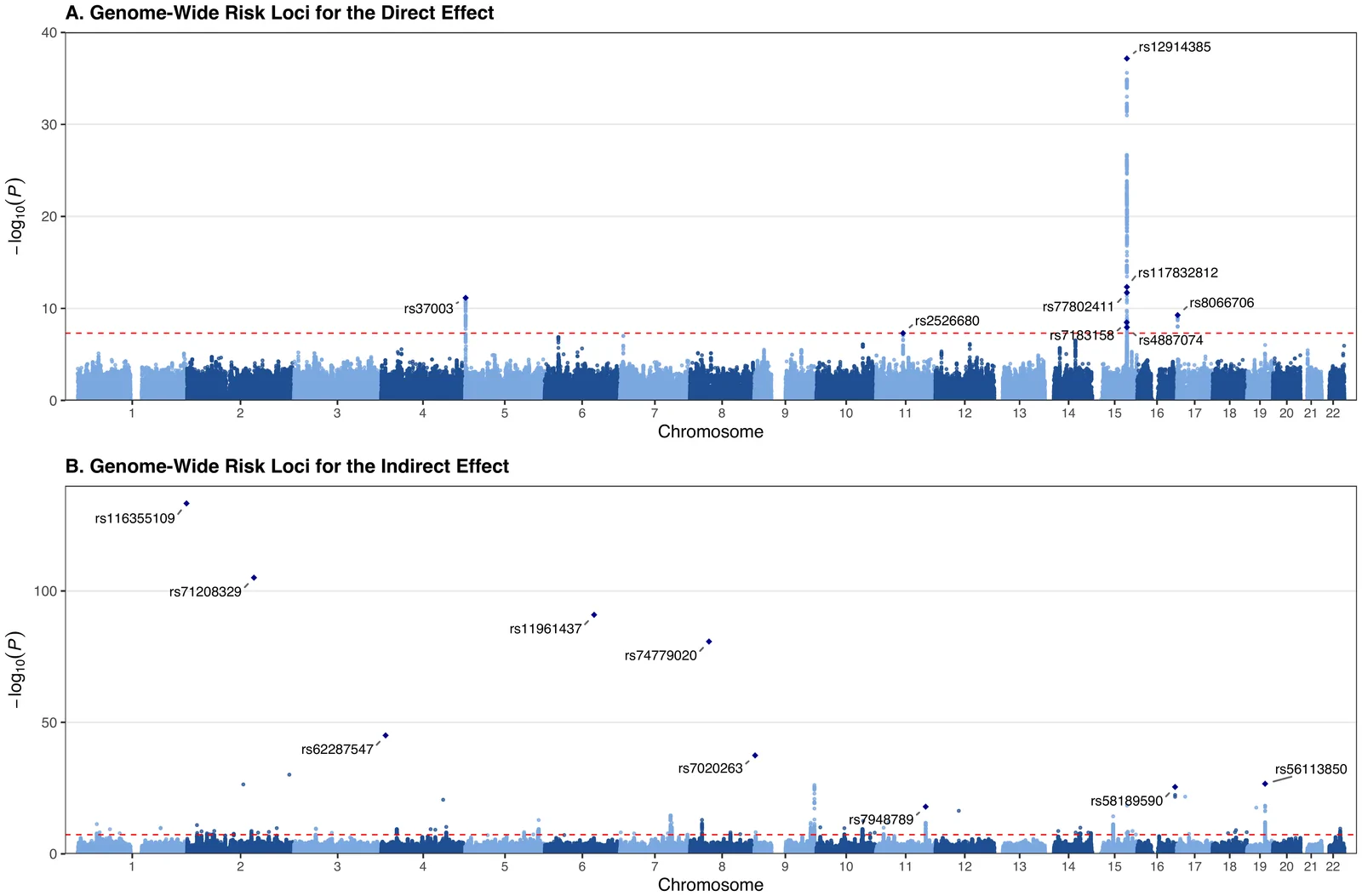
Component-specific genome-wide association results for **(A)** the direct component statistically independent of measured current smoking and **(B)** the model-defined indirect component associated with measured current smoking. The y axis shows −log_10_(P) and the red dashed line marks *P* = 5×10^-8^.

**Figure 4:**
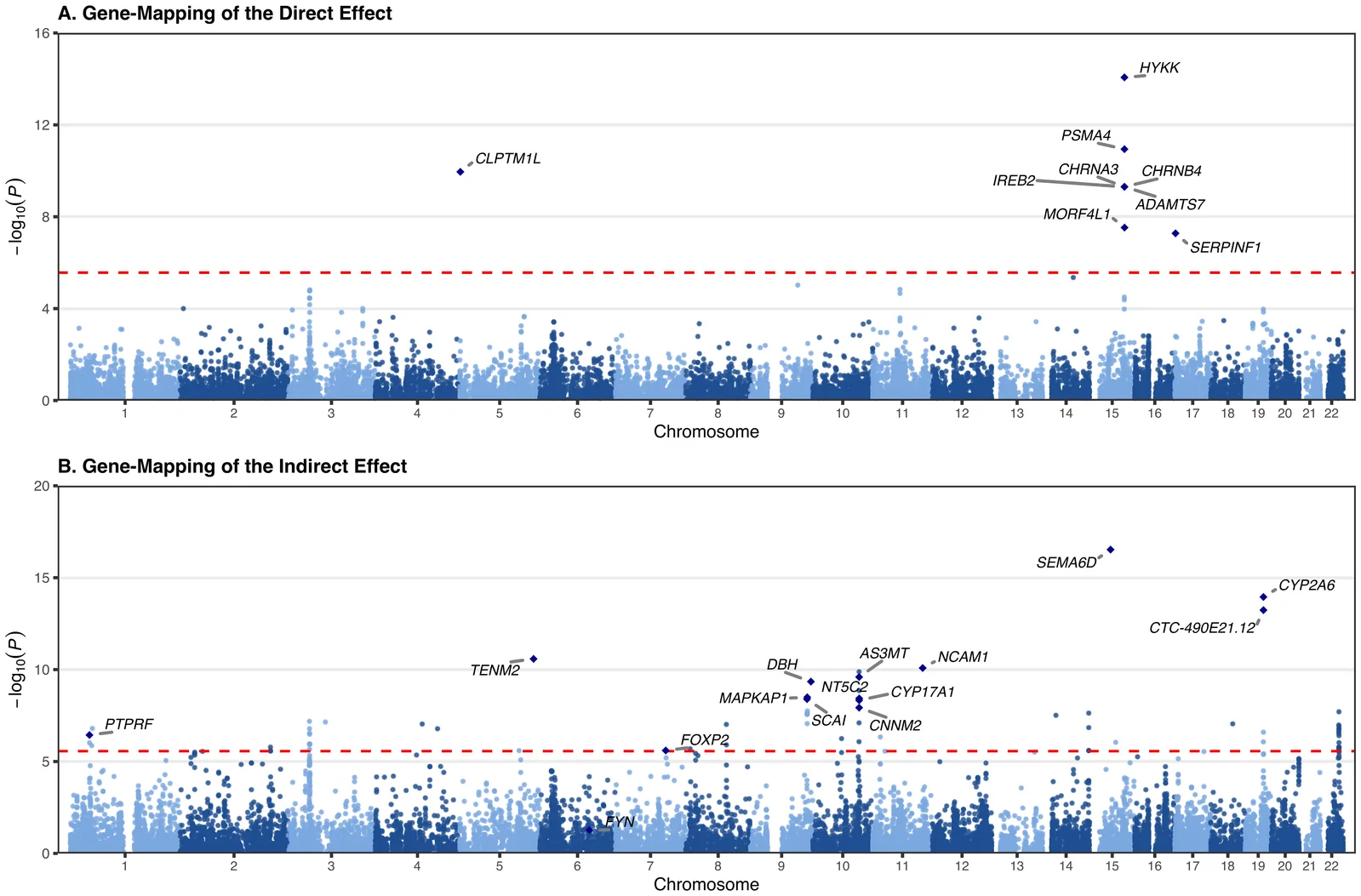
Gene-based association results for the **(A)** direct and **(B)** indirect components. The y axis shows −log_10_(P), and the red dashed line marks the gene-level significance threshold.

The component-specific analyses revealed different architectures (**Figure 3**). The direct-component analysis was dominated by concentrated signals on chromosomes 5 and 15, whereas the indirect-component analysis was highly polygenic. The strongest direct-component locus fell within the chromosome 15q25 nicotinic-receptor genes, with additional loci on chromosomes 5, 11 and 17 (**Supplementary Table 3**). Functional annotation of the indirect-component variants highlighted several high-impact substitutions, including a nonsynonymous variant in *KIAA1109* (**Supplementary Table 4** and **Supplementary Figure 1**). Gene-level analysis and expression-based fine-mapping prioritised, for the direct component statistically independent of measured current smoking, candidate genes including *CLPTM1L*, *IREB2*, *HYKK*, *PSMA4*, *MORF4L1* and *ADAMTS7*, and for the model-defined indirect component associated with measured current smoking a larger set including *AS3MT*, *VRK2*, *PIK3C2A* and *ARID5B*, consistent with the genetics of smoking behaviour (**Figure 4**; **Supplementary Tables 5**, **6**).

### 3.4 Divergent tissue and pathway enrichment

**Figure 5:**
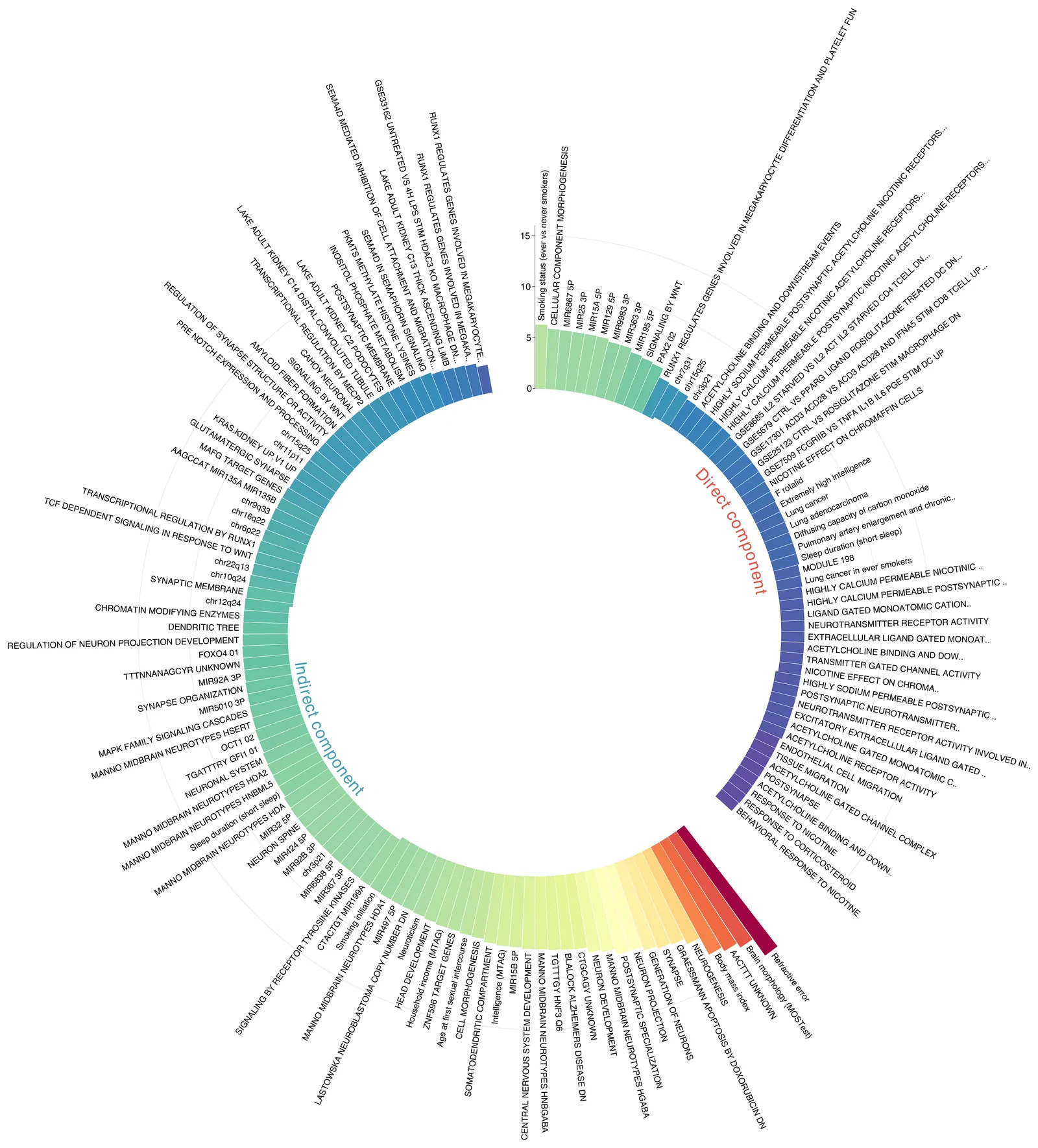
Gene-set enrichment for genes in the direct and indirect components. Bars are arranged around the circular plot by component, with bar height representing −log_10_(P); the curved labels identify the direct and indirect components. Direct-component terms emphasize carcinogenesis and cellular pathways, whereas indirect-component terms emphasize neuronal and behavioural pathways.

The two components showed contrasting biology (**Figure 5**; **Supplementary Figure 2**). Direct-component genes were enriched in lung tissue (Bonferroni-adjusted *P* < 1 × 10^-^³) and in cerebellar and cortical brain regions, and in carcinogenesis-related pathways together with acetylcholine-receptor activity. Indirect-component genes were enriched in brain regions (Bonferroni-adjusted *P* < 1×10^-9^) and in neuronal and synaptic pathways, and overlapped catalogued traits for smoking initiation and nicotine dependence.

### 3.5 CLPTM1L expression responds to non-nicotine pathological stress

**Figure 6:**
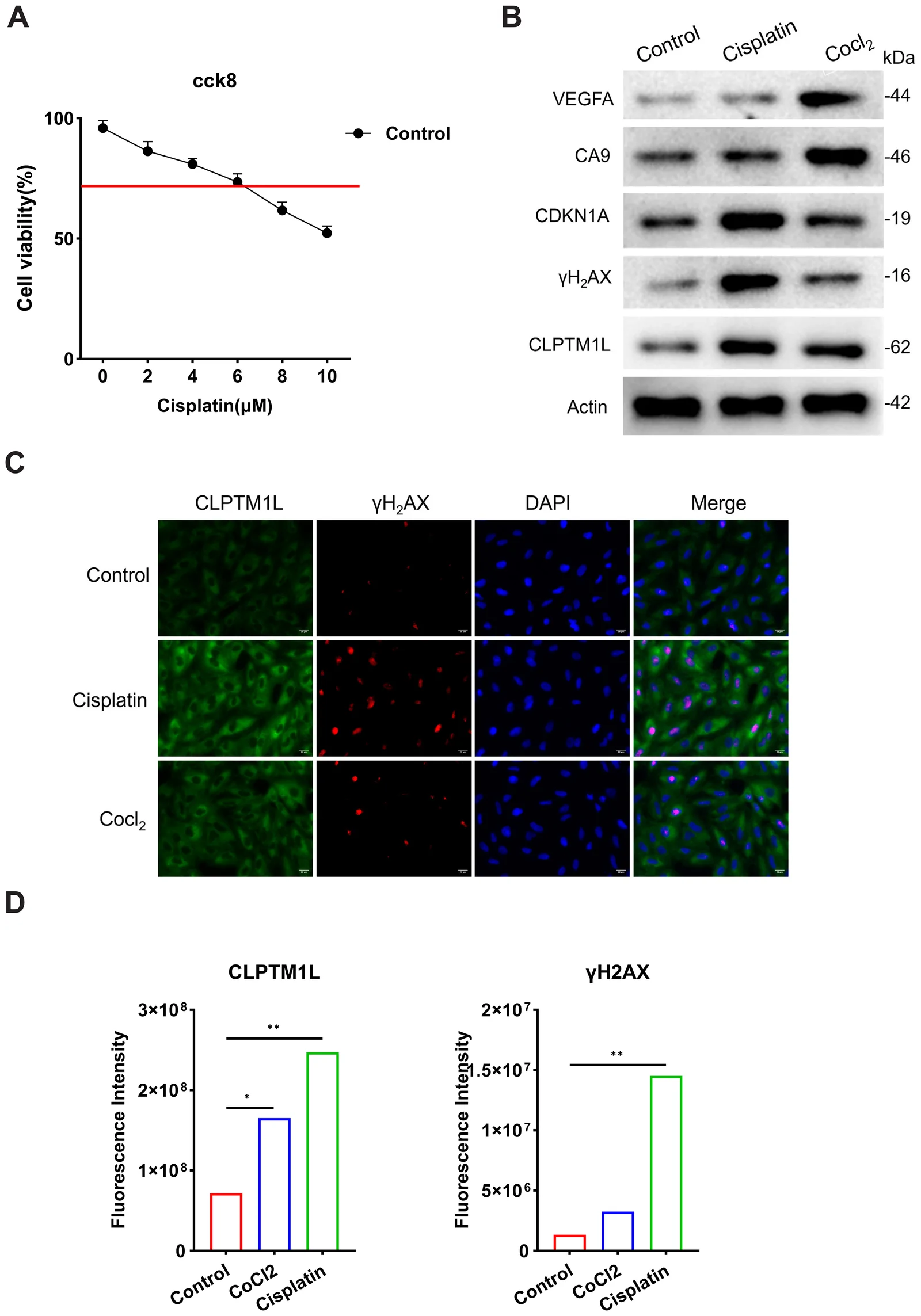
CLPTM1L expression responds to non-nicotine pathological stress in A549 lung adenocarcinoma cells. **(A)** CCK-8 titration of cisplatin selecting a sub-lethal 6 µM dose. **(B)** Western blot for CLPTM1L, the genotoxic markers γH2AX and CDKN1A, the hypoxia markers CA9 and VEGFA, and β-actin across control, cisplatin and cobalt chloride conditions. **(C)** Immunofluorescence for CLPTM1L (green), γH2AX (red) and DAPI (blue). **(D)** ImageJ quantification of CLPTM1L and γH2AX mean fluorescence intensity. Data are three independent biological replicates, paired t test, *P < 0.05, **P < 0.01.

*CLPTM1L* responded to non-nicotine pathological stress in lung adenocarcinoma cells (**Figure 6**). Cisplatin titration identified 6 µM as a sub-lethal dose retaining about 73% viability at 24 h (**Figure 6A**). Cisplatin increased the DNA-damage markers γH2AX and CDKN1A and cobalt chloride increased the hypoxia markers CA9 and VEGFA, providing positive-control evidence that the intended cell-state markers responded (**Figure 6B**). In the same experiments, CLPTM1L protein increased under both stresses relative to vehicle, with a stronger rise under genotoxic stress, and showed a band at the expected molecular weight (**Figure 6B**). Immunofluorescence localised CLPTM1L to the cytoplasm and perinuclear region and showed concordant induction, with mean *CLPTM1L* intensity about 3.4-fold higher under cisplatin and about 2.3-fold higher under cobalt chloride than control (**Figures 6C, D**). The γH2AX signal rose about 11-fold under cisplatin but was unchanged under cobalt chloride (**Figure 6D**). All comparisons used three independent biological replicates with paired t tests at *P* < 0.05. These data indicate that *CLPTM1L* expression is responsive in a disease-relevant non-nicotine cell state, and they do not address whether *CLPTM1L* is associated with NSCLC risk.

### 3.6 Suggestive innate immune and inflammatory signals

Across the six traits examined together, the NSCLC association signal localised alongside those of ADHD, smoking, C-reactive protein, asthma and neuroticism at loci assigned to the direct component, including the chromosome 15q25 nicotinic-receptor cluster and the 5p15.33/*CLPTM1L* region. *TRAF3IP2* and *TRIM8* were prioritised in separate genome-wide gene-level analyses (**Figure 1A**). At the level of systemic inflammation, ADHD and NSCLC were each positively genetically correlated with circulating C-reactive protein, an acute-phase biomarker (ADHD rg=0.26, SE = 0.036, *P* = 8.2×10^-^¹³; NSCLC rg=0.13, SE = 0.049, *P* = 0.0074; **Figure 1B**), consistent with shared genetic architecture involving an acute-phase biomarker. Associations with the additional inflammatory traits were exploratory.

At the pathway level, MAGMA gene-set analysis of the NSCLC gene-level signal (not the direct/indirect component statistics) nominated innate pattern-recognition-receptor and NF-κB/antiviral programmes. RIG-I-like receptor signalling ranked fifth among 16,785 gene sets (*P* = 5.6×10^-5^), with nominal enrichment of NOD-like and Toll-like receptor signalling, TRAF6-mediated induction, and chemokine and cytokine-receptor pathways (**Figure 1C**). The prioritised candidate genes included the innate regulators *TRAF3IP2*, an interleukin-17-to-NF-κB adaptor, and *TRIM8*, a Toll-like-receptor and NF-κB regulator. These enrichments were nominal and did not survive global Benjamini-Hochberg correction. The q=0.017 value applies only to the innate-pathway subset.

## 4 Discussion

The genetic relationship between ADHD and NSCLC in the ADHD-anchored model yielded approximate model-based partitions of genetic covariance: about 62% associated with the measured current-smoking phenotype and about 38% not captured by that phenotype. These values are not precise biological proportions. This suggests that the observed genetic covariance is not fully captured by the measured current-smoking phenotype, but it does not exclude other smoking-related exposures or establish a causal route. These are partitions of genetic covariance rather than proof of causation, and the central interpretation of a direct component will require triangulation as outlined below.

The indirect component associated with measured current smoking was polygenic and brain-enriched, consistent with genetic links between ADHD-related liability and smoking and with observational evidence of psychiatric comorbidity and smoking (22, 23). Genes prioritised here, including the nicotine-metabolism gene *AS3MT*, are consistent with the known genetics of nicotine dependence and smoking initiation (24). These findings can be situated within the recent five-factor cross-disorder analysis of fourteen psychiatric disorders (25); however, our two-indicator specification is ADHD-anchored and exploratory, and we do not interpret it as a general psychopathology or p-factor construct.

The direct component is novel as a covariance partition, not as locus discovery. The 5p15.33 and 15q25 regions are established lung-cancer and smoking-related loci; our contribution is to partition their association within an ADHD-anchored model, not to claim discovery of new loci or unique attribution to ADHD. The chromosome 15q25 nicotinic-receptor locus contributed to both components with distinct functional implications, echoing earlier mediation work in which 15q25 variants retained a direct effect on lung cancer after adjustment for smoking (26). The enrichment of acetylcholine-receptor activity is consistent with a possible cholinergic signal in lung tissue, but does not establish a conditional nicotinic mechanism (27), and experimental evidence in smoke-exposed epithelium provides context but does not establish the direct component here (28).

*CLPTM1L* responded to two non-nicotine pathological stresses in lung adenocarcinoma cells, which is consistent with, but does not validate, its prioritisation in the direct component. *CLPTM1L* lies in the 5p15.33 region associated with lung adenocarcinoma in never-smokers, and the protein restrains genotoxic-stress apoptosis and is required for Ras-driven lung tumourigenesis (29–31). Our experiment adds only that *CLPTM1L* expression is engaged by genotoxic and hypoxic stress without nicotine, and the stronger induction under genotoxic stress is consistent with that described role. It does not establish a causal or oncogenic function, which would require loss-of-function and rescue studies. *IREB2*, another prioritised candidate in the direct component, regulates iron homeostasis with prognostic relevance in NSCLC (32).

The CRP associations support a shared genetic component involving an acute-phase biomarker; pathway and cell-count findings remain hypothesis-generating. At the pathway level the NSCLC signal showed nominal enrichment of pattern-recognition-receptor and NF-κB/antiviral programmes, with candidate genes including *TRAF3IP2* and *TRIM8*. The chromosome 15q25 locus has been discussed in relation to cholinergic anti-inflammatory signalling, but our summary-statistics analysis does not test that mechanism (28). This locus therefore remains a hypothesis for functional follow-up rather than evidence of an established cholinergic–innate immune interface.

These distinctions may motivate hypotheses for integrated prevention. The smoking-associated component could be considered alongside interventions that address psychiatric symptoms and smoking together (33, 34), whereas the conditional component not captured by measured current smoking should not be interpreted as evidence that smoking-focused prevention is insufficient, because other smoking-related exposures and confounding remain possible; genetic risk stratification may be evaluated in future studies but is not a clinical screening recommendation (35). Psychiatric comorbidity is common among patients with cancer, which makes such integrated care practical (36–38).

This study has limitations. First, the shared component is ADHD-anchored; neuroticism did not survive correction and was exploratory, so the findings do not represent general psychopathology. Second, the Genomic-SEM partition is conditional on the measured current-smoking phenotype and does not establish causation; the A549 assay is an expression-level observation in a single adenocarcinoma line and did not include knockdown, overexpression or rescue. Third, the core datasets were predominantly European-ancestry and European LD scores were used; some screening resources were not uniformly ancestry-matched, so cross-ancestry heterogeneity and LD mismatch remain possible. Fourth, smoking was the only modelled mediator; other tobacco exposures, socioeconomic factors and comorbidities may also contribute (4). Fifth, the innate signal was detected at the pathway and systemic-biomarker level and was nominal after correction across 16,785 gene sets; CRP/immune-marker correlations were exploratory and do not establish cell-specific mechanisms. Cell-type-resolved and functional studies are needed. Participant-level identifiers were not available in the public summary statistics, so sample overlap among public GWAS sources could not be assessed directly. Source-specific sample-size definitions and coordinate builds are documented in **Supplementary Table 1**. Software versions and analysis parameters are reported in the Methods.

## 5 Conclusion

The genetic covariance shared between ADHD and NSCLC included an approximate component statistically independent of the measured current-smoking phenotype, with candidate genes showing dual lung and brain enrichment. *CLPTM1L*, a prioritised candidate gene in this component, showed expression responsiveness to non-nicotine pathological stress in lung adenocarcinoma cells. Pending causal and functional confirmation, these results nominate a genetic lung-brain association for the traits studied rather than an established causal pathway. The innate pathway enrichments are suggestive, not established mechanisms.

## Data Availability

The original contributions presented in the study are included in the article/**Supplementary Material**, further inquiries can be directed to the corresponding author/s.
