# Supplementary material for: ADHD-anchored partitioning of non-small cell lung cancer genetic covariance identifies CLPTM1L and suggestive innate-immune signals
Source: Front Immunol. 2026 Aug 13;17:1913958. doi: 10.3389/fimmu.2026.1913958 (PMC13518126; doi:10.3389/fimmu.2026.1913958)
Supplement: Supplementary file 1 [file DataSheet1.docx]

Table S1. GWAS data sources and metadata

| **Abbreviation** | **Trait / source study** | **Exact file / accession used** | **Source-reported sample size and ancestry** | **Genome build** | **PMID** | **DOI and stable download / metadata URL** |
| --- | --- | --- | --- | --- | --- | --- |
| ADHD | Attention-deficit/hyperactivity disorder; PGC (Demontis et al., 2023) | ADHD2022_iPSYCH_deCODE_PGC.meta.gz; Figshare 22564390/file 40036684 | 38,691 cases + 186,843 controls; predominantly European | GRCh37/hg19 | 36702997 | 10.1038/s41588-022-01285-8; https://ndownloader.figshare.com/files/40036684 |
| ALZ | Alzheimer's disease (biological father: Alzheimer’s/other dementia); MVP/VA (Verma et al., 2024) | GCST90475249.tsv.gz; EBI accession GCST90475249 | 19,600 cases + 296,068 controls (N=315,668); European | GRCh38 | 39024449 | 10.1126/science.adj1182; file https://ftp.ebi.ac.uk/pub/databases/gwas/summary_statistics/GCST90475001-GCST90476000/GCST90475249/GCST90475249.tsv.gz; metadata https://ftp.ebi.ac.uk/pub/databases/gwas/summary_statistics/GCST90475001-GCST90476000/GCST90475249/GCST90475249.tsv.gz-meta.yaml |
| ANX | Panic disorder; PGC (Forstner et al., 2019; issue publication 2021) | pgc-panic2019.vcf.tsv.gz; Figshare 16602218/file 30731276 | 2,147 cases + 7,760 controls; 6 cohorts; European/1000 Genomes Phase 1 | GRCh37 | 31712720 | 10.1038/s41380-019-0590-2; https://ndownloader.figshare.com/files/30731276 |
| ASD | Autism; ADuLT/Danish cohort (Pedersen et al., 2023) | GCST90275152.tsv; EBI accession GCST90275152 | 18,235 cases + 36,741 controls (N=54,976); European | GRCh37 | 37689771 | 10.1038/s41467-023-41210-z; file https://ftp.ebi.ac.uk/pub/databases/gwas/summary_statistics/GCST90275001-GCST90276000/GCST90275152/GCST90275152.tsv; metadata https://ftp.ebi.ac.uk/pub/databases/gwas/summary_statistics/GCST90275001-GCST90276000/GCST90275152/GCST90275152.tsv-meta.yaml |
| BIP | Bipolar disorder; O’Connell et al. (2025) | bip2024_multianc_no23andMe.gz; Figshare 27216117/file 49760775 | 158,036 cases + 2,796,499 controls; multi-ancestry (EUR/AFR/EAS/LAT); variant-level N varies | GRCh37/hg19 | 39843750 | 10.1038/s41586-024-08468-9; https://ndownloader.figshare.com/files/49760775 |
| AN | Anorexia nervosa; PGC (Watson et al., 2019; source file label AN/ED) | pgcAN2.2019-07.vcf.tsv.gz; Figshare 14671980/file 28169271 | 16,992 cases + 55,525 controls; predominantly European; 33 cohorts | GRCh37 | 31308545 | 10.1038/s41588-019-0439-2; https://ndownloader.figshare.com/files/28169271 |
| MDD | Postpartum depression (PPD; analysis label MDD); PGC (Guintivano et al., 2023) | ppd2023_EUR_sumstats.tsv.gz; Figshare 24204843/file 42479649 | 17,339 cases + 53,426 controls; 18 cohorts; European | GRCh37/hg19 | 37849304 | 10.1176/appi.ajp.20230053; https://ndownloader.figshare.com/files/42479649 |
| TS | Tourette syndrome; PGC (Yu et al., 2019) | TS_Oct2018.gz; Figshare 14672232/file 28169940 | 4,819 cases + 9,488 controls; European | GRCh37/hg19 | 30818990 | 10.1176/appi.ajp.2018.18070857; https://ndownloader.figshare.com/files/28169940 |
| OCD | Obsessive-compulsive disorder; OCD-3 (Strom et al., 2025) | daner_OCD_full_wo23andMe_190522.gz; Figshare 28707155/file 53357735 | 23,493 cases + 1,114,613 controls; European | GRCh37 | 40360802 | 10.1038/s41588-025-02189-z; https://ndownloader.figshare.com/files/53357735 |
| PTSD | Post-traumatic stress disorder; PGC African-ancestry GWAS (Nievergelt et al., 2024) | aam_ptsd_pcs_v5_jan4_2022.vcf.gz; Figshare 26349322/file 47850136 | 11,560 cases + 39,474 controls; African ancestry | GRCh37; HRC v1.0 imputation reference | 38637617 | 10.1038/s41588-024-01707-9; https://ndownloader.figshare.com/files/47850136 |
| SCZ | Schizophrenia; PGC3 (Trubetskoy et al., 2022) | PGC3_SCZ_wave3.core.autosome.public.v3.vcf.tsv.gz; Figshare 19426775/file 34517807 | Header N=64,322 cases + 90,947 controls; primarily European + East Asian; variant-level N varies | GRCh37; HRC v1.1 imputation reference | 35396580 | 10.1038/s41586-022-04434-5; https://ndownloader.figshare.com/files/34517807 |
| Neuroticism | Neuroticism; EBI GCST90029028 (catalogue publication metadata: Loh et al., 2018) | GCST90029028_buildGRCh37.tsv; EBI accession GCST90029028 | N=393,411; European (variant-level n=372,066) | GRCh37 | 29892013 | catalogue publication metadata DOI 10.1038/s41588-018-0144-6; file https://ftp.ebi.ac.uk/pub/databases/gwas/summary_statistics/GCST90029001-GCST90030000/GCST90029028/GCST90029028_buildGRCh37.tsv; metadata https://ftp.ebi.ac.uk/pub/databases/gwas/summary_statistics/GCST90029001-GCST90030000/GCST90029028/GCST90029028_buildGRCh37.tsv-meta.yaml |
| Smoking | Current tobacco smoking; UK Biobank / MRC-IEU OpenGWAS (ukb-b-223; 2018) | OpenGWAS accession ukb-b-223 (source file was requested through the OpenGWAS API) | N=462,434; European (source-reported; variant-level N may vary) | GRCh37/HG19 | NR | Stable dataset page: https://gwas.mrcieu.ac.uk/datasets/ukb-b-223/ |
| NSCLC | Non-small-cell lung cancer; FinnGen release 12 (Kurki et al., 2023) | finngen_R12_C3_LUNG_NONSMALL_EXALLC.gz; endpoint C3_LUNG_NONSMALL_EXALLC | 6,446 cases + 378,749 controls (N=385,195); Finnish/European FinnGen participants | GRCh38/hg38 | 36653562 | 10.1038/s41586-022-05473-8; file https://storage.googleapis.com/finngen-public-data-r12/summary_stats/release/finngen_R12_C3_LUNG_NONSMALL_EXALLC.gz; manifest https://storage.googleapis.com/finngen-public-data-r12/summary_stats/finngen_R12_manifest.tsv |
| CRP | C-reactive protein levels; Said et al. (2022) | GCST90029070_buildGRCh37.tsv.gz; EBI accession GCST90029070 | N=575,531; European | GRCh37 | 35459240 | 10.1038/s41467-022-29650-5; file https://ftp.ebi.ac.uk/pub/databases/gwas/summary_statistics/GCST90029001-GCST90030000/GCST90029070/GCST90029070_buildGRCh37.tsv.gz; metadata https://ftp.ebi.ac.uk/pub/databases/gwas/summary_statistics/GCST90029001-GCST90030000/GCST90029070/GCST90029070_buildGRCh37.tsv.gz-meta.yaml |
| ASTHMA | Asthma; Demenais et al. (2018) | 29273806-GCST006862-EFO_0000270-build37.f.tsv.gz; EBI accession GCST006862 | 19,954 European-ancestry cases + 107,715 European-ancestry controls (N=127,669); exploratory comparator | GRCh37/hg19 formatted file; EBI harmonised metadata GRCh38 | 29273806 | 10.1038/s41588-017-0014-7; file https://ftp.ebi.ac.uk/pub/databases/gwas/summary_statistics/GCST006001-GCST007000/GCST006862/harmonised/29273806-GCST006862-EFO_0000270-build37.f.tsv.gz; metadata https://ftp.ebi.ac.uk/pub/databases/gwas/summary_statistics/GCST006001-GCST007000/GCST006862/harmonised/29273806-GCST006862-EFO_0000270.h.tsv.gz-meta.yaml; study https://www.ebi.ac.uk/gwas/rest/api/studies/GCST006862 |
| EOS | Eosinophil count; Astle et al. (2016) | eo_N172275_narrow_form.tsv.gz; EBI accession GCST004606 | N=172,275; European; exploratory comparator | GRCh37 | 27863252 | 10.1016/j.cell.2016.10.042; file https://ftp.ebi.ac.uk/pub/databases/gwas/summary_statistics/GCST004001-GCST005000/GCST004606/eo_Jan2018_update/eo_N172275_narrow_form.tsv.gz; README https://ftp.ebi.ac.uk/pub/databases/gwas/summary_statistics/GCST004001-GCST005000/GCST004606/eo_Jan2018_update/README.txt; study https://www.ebi.ac.uk/gwas/rest/api/studies/GCST004606 |
| LYMPH | Lymphocyte count; Astle et al. (2016) | 27863252-GCST004627-EFO_0004587-Build37.f.tsv.gz; EBI accession GCST004627 | N=171,643; European; exploratory comparator | GRCh37 formatted file; EBI harmonised metadata GRCh38 | 27863252 | 10.1016/j.cell.2016.10.042; file https://ftp.ebi.ac.uk/pub/databases/gwas/summary_statistics/GCST004001-GCST005000/GCST004627/harmonised/27863252-GCST004627-EFO_0004587-Build37.f.tsv.gz; metadata https://ftp.ebi.ac.uk/pub/databases/gwas/summary_statistics/GCST004001-GCST005000/GCST004627/harmonised/27863252-GCST004627-EFO_0004587.h.tsv.gz-meta.yaml; study https://www.ebi.ac.uk/gwas/rest/api/studies/GCST004627 |
| WBC | White-blood-cell count; Astle et al. (2016) | wbc_N172435_narrow_form.tsv.gz; Sanger/Blood Cell Genetics directory; EBI accession GCST004610 | N=172,435; European | GRCh37 | 27863252 | 10.1016/j.cell.2016.10.042; https://ftp.sanger.ac.uk/pub/project/humgen/summary_statistics/human/2017-12-12/hematological_traits/wbc/wbc_N172435_narrow_form.tsv.gz; EBI harmonised file https://ftp.ebi.ac.uk/pub/databases/gwas/summary_statistics/GCST004001-GCST005000/GCST004610/harmonised/27863252-GCST004610-EFO_0004308-Build37.f.tsv.gz; metadata https://ftp.ebi.ac.uk/pub/databases/gwas/summary_statistics/GCST004001-GCST005000/GCST004610/harmonised/27863252-GCST004610-EFO_0004308.h.tsv.gz-meta.yaml; study https://www.ebi.ac.uk/gwas/rest/api/studies/GCST004610 |
| MONO | Monocyte count; Astle et al. (2016) | mono_N170721_narrow_form.tsv.gz; Sanger/Blood Cell Genetics directory; EBI accession GCST004625 | N=170,721; European | GRCh37 | 27863252 | 10.1016/j.cell.2016.10.042; https://ftp.sanger.ac.uk/pub/project/humgen/summary_statistics/human/2017-12-12/hematological_traits/mono/mono_N170721_narrow_form.tsv.gz; EBI harmonised file https://ftp.ebi.ac.uk/pub/databases/gwas/summary_statistics/GCST004001-GCST005000/GCST004625/harmonised/27863252-GCST004625-EFO_0005091-Build37.f.tsv.gz; metadata https://ftp.ebi.ac.uk/pub/databases/gwas/summary_statistics/GCST004001-GCST005000/GCST004625/harmonised/27863252-GCST004625-EFO_0005091.h.tsv.gz-meta.yaml; study https://www.ebi.ac.uk/gwas/rest/api/studies/GCST004625 |

Verification note: Table S1 lists the accession, exact file name, source-reported sample size, ancestry, genome build and stable provider link for each dataset. “NR” indicates information not reported by the source. Study-level sample sizes are reported as recorded by the data providers; variant-level N may vary where indicated.

Table S2. LDSC bivariate genetic correlations between 12 psychiatric/personality traits and NSCLC, with Benjamini-Hochberg FDR across the 11 interpretable traits. ADHD is the only trait surviving FDR<0.05 (q=2.6e-4); neuroticism is nominal (raw P=0.015, q=0.056) and retained only as a secondary exploratory indicator. Anorexia nervosa, OCD and Tourette syndrome show NEGATIVE correlations and were not used as positive indicators of the ADHD-anchored model. Panic disorder (ANX) gave an out-of-bounds heritability estimate (not interpretable).

| **Trait (Full name)** | **Abbr.** | **rg vs NSCLC** | **SE** | **Raw P** | **FDR-adj P (BH)** |
| --- | --- | --- | --- | --- | --- |
| Attention-Deficit/Hyperactivity Disorder | ADHD | 0.2448 | 0.0579 | 2.37e-05 | 2.6e-04 |
| Tourette Syndrome | TS | -0.2655 | 0.1085 | 0.0144 | 0.056 |
| Neuroticism | Neuroticism | 0.1261 | 0.0520 | 0.0153 | 0.056 |
| Anorexia Nervosa | AN | -0.1705 | 0.0783 | 0.0294 | 0.065 |
| Obsessive-Compulsive Disorder | OCD | -0.1511 | 0.0688 | 0.0280 | 0.065 |
| Major Depressive Disorder (Postpartum) | MDD | 0.1443 | 0.1004 | 0.1504 | 0.276 |
| Post-Traumatic Stress Disorder | PTSD | 0.2072 | 0.1635 | 0.2052 | 0.282 |
| Alzheimer's Disease | ALZ | -0.3104 | 0.2396 | 0.1951 | 0.282 |
| Bipolar Disorder | BIP | -0.0484 | 0.0548 | 0.3770 | 0.377 |
| Autism Spectrum Disorder | ASD | 0.0679 | 0.0757 | 0.3693 | 0.377 |
| Schizophrenia | SCZ | 0.0398 | 0.0422 | 0.3452 | 0.377 |
| Panic Disorder | ANX | NA | NA | NA | NA |

Table S3. Lead SNP Annotation

| **GWAS** | **rsID** | **uniqID** | **chr** | **pos** | **p** | **CADD** | **RDB** | **symbol** | **annot** | **dist** | **nIndSigSNPs** | **IndSigSNPs** |
| --- | --- | --- | --- | --- | --- | --- | --- | --- | --- | --- | --- | --- |
| Direct effect | rs37003 | 5:1356771:A:C | 5 | 1356771 | 7.12730389466e-12 | 2.959 | NA | RP11-325I22.2 | intergenic | 6926 | 4 | rs37003;rs2736109;rs6554758;rs35953391 |
| Direct effect | rs2526680 | 11:61611216:C:T | 11 | 61611216 | 4.93036966797e-08 | 0.015 | NA | FADS2 | intronic | 0 | 1 | rs2526680 |
| Direct effect | rs7183158 | 15:78546903:A:G | 15 | 78546903 | 3.26886452162e-09 | 0.379 | 5 | RP11-762H8.3 | ncRNA_intronic | 0 | 1 | rs7183158 |
| Direct effect | rs12914385 | 15:78898723:C:T | 15 | 78898723 | 6.80411605975e-38 | 0.496 | 7 | CHRNA3 | intronic | 0 | 13 | rs12914385;rs3825845;rs67175876;rs67426328;rs11633178;rs12905273;rs12913260;rs7403155;rs34184420;rs2869045;rs17483721;rs11637656;rs56117933 |
| Direct effect | rs117832812 | 15:78912472:A:C | 15 | 78912472 | 4.6904885846e-13 | 3.844 | 4 | CHRNA3 | intronic | 0 | 8 | rs117832812;rs67175876;rs11072774;rs74634068;rs2869045;rs11637656;rs376499689;rs3825845 |
| Direct effect | rs77802411 | 15:78925414:C:T | 15 | 78925414 | 1.93933819346e-12 | 0.386 | 5 | CHRNB4 | intronic | 0 | 1 | rs77802411 |
| Direct effect | rs4887074 | 15:78952110:C:G | 15 | 78952110 | 1.14092587935e-08 | 7.268 | 5 | CHRNB4 | intronic | 0 | 10 | rs4887074;rs11072774;rs74634068;rs12913260;rs2869045;rs376499689;rs12443170;rs3825845;rs67175876;rs11633178 |
| Direct effect | rs8066706 | 17:1692140:C:T | 17 | 1692140 | 5.37227228649e-10 | 4.779 | 5 | SMYD4 | intronic | 0 | 2 | rs8066706;rs1894286 |
| Indirect effect | rs3001723 | 1:44037685:A:G | 1 | 44037685 | 5.0520538452e-12 | 2.712 | 5 | PTPRF | intronic | 0 | 2 | rs3001723;rs2782640 |
| Indirect effect | rs3905052 | 1:49463925:A:G | 1 | 49463925 | 4.5868408233e-08 | 4.431 | 6 | AGBL4 | intronic | 0 | 1 | rs3905052 |
| Indirect effect | rs12118183 | 1:50537618:C:T | 1 | 50537618 | 6.83010600933e-09 | 15.84 | 7 | ELAVL4 | intronic | 0 | 1 | rs12118183 |
| Indirect effect | rs11210229 | 1:73860028:A:G | 1 | 73860028 | 4.56579828083e-10 | 3.114 | 6 | RP4-598G3.1 | intergenic | 39094 | 1 | rs11210229 |
| Indirect effect | rs6593680 | 1:96169760:A:G | 1 | 96169760 | 1.49102432575e-08 | 2.531 | 7 | RP11-286B14.1 | ncRNA_intronic | 0 | 1 | rs6593680 |
| Indirect effect | rs56334517 | 1:189083888:C:T | 1 | 189083888 | 1.94887093401e-10 | 2.105 | 6 | RP11-373J16.2 | intergenic | 17593 | 1 | rs56334517 |
| Indirect effect | rs116355109 | 1:247864269:C:T | 1 | 247864269 | 4.93760134568e-134 | 3.384 | 6 | RP11-634B7.4 | ncRNA_intronic | 0 | 1 | rs116355109 |
| Indirect effect | rs4557006 | 2:22443840:A:G | 2 | 22443840 | 1.20355755732e-11 | 11.02 | 7 | AC068490.2 | ncRNA_intronic | 0 | 2 | rs4557006;rs2339515 |
| Indirect effect | rs12469094 | 2:22967228:A:C | 2 | 22967228 | 1.21200722827e-08 | 6.269 | 6 | RN7SKP27 | intergenic | 117196 | 1 | rs12469094 |
| Indirect effect | rs6723226 | 2:32849207:A:G | 2 | 32849207 | 2.91364694863e-09 | 2.521 | 7 | TTC27 | intergenic | 3892 | 3 | rs6723226;rs805831;rs479333 |
| Indirect effect | rs1004787 | 2:45159091:A:G | 2 | 45159091 | 1.61780794304e-08 | 1.499 | 4 | RP11-89K21.1 | ncRNA_intronic | 0 | 1 | rs1004787 |
| Indirect effect | rs1985832 | 2:51198675:C:T | 2 | 51198675 | 3.94026842696e-08 | 2.378 | NA | NRXN1 | intronic | 0 | 1 | rs1985832 |
| Indirect effect | rs3087898 | 2:61765074:A:G | 2 | 61765074 | 2.05216605738e-09 | 18.28 | NA | XPO1 | UTR5 | 0 | 1 | rs3087898 |
| Indirect effect | rs28545614 | 2:105994827:C:T | 2 | 105994827 | 2.42587660048e-08 | 0.321 | NA | AC012360.6 | ncRNA_exonic | 0 | 1 | rs28545614 |
| Indirect effect | rs56200757 | 2:127729252:C:T | 2 | 127729252 | 3.97957066618e-27 | 4.701 | 2b | RNU7-182P | intergenic | 45745 | 1 | rs56200757 |
| Indirect effect | rs275160 | 2:145967878:C:T | 2 | 145967878 | 4.8179848682e-09 | 0.283 | NA | TEX41 | intergenic | 27662 | 1 | rs275160 |
| Indirect effect | rs6727997 | 2:146346285:A:G | 2 | 146346285 | 1.98164774977e-09 | 0.486 | 7 | AC092484.1 | upstream | 0 | 4 | rs6727997;rs72863305;rs10193706;rs953246 |
| Indirect effect | rs71208329 | 2:151915828:C:T | 2 | 151915828 | 8.83039588763e-106 | 3.707 | 5 | AC023469.1 | intergenic | 10540 | 1 | rs71208329 |
| Indirect effect | rs72970054 | 2:161356939:A:T | 2 | 161356939 | 9.2012601838e-09 | 0.95 | 6 | RBMS1 | intergenic | 6634 | 1 | rs72970054 |
| Indirect effect | rs263771 | 2:185921692:A:C | 2 | 185921692 | 4.58059556136e-09 | 0.141 | 6 | ZNF804A | intergenic | 117473 | 2 | rs263771;rs2046308 |
| Indirect effect | rs12693975 | 2:203720745:A:G | 2 | 203720745 | 1.67508446676e-10 | 0.181 | 7 | ICA1L | intronic | 0 | 2 | rs12693975;rs72938315 |
| Indirect effect | rs16825937 | 2:232290893:A:G | 2 | 232290893 | 7.77711630044e-31 | 0.774 | 4 | ZBTB8OSP2 | intergenic | 7741 | 1 | rs16825937 |
| Indirect effect | rs4955417 | 3:49298205:C:T | 3 | 49298205 | 2.3163125155e-10 | 11.88 | 1f | RP11-3B7.1 | exonic | 0 | 1 | rs4955417 |
| Indirect effect | rs7613360 | 3:49916710:C:T | 3 | 49916710 | 3.98295854112e-10 | 2.031 | 5 | ACTBP13 | intergenic | 1297 | 2 | rs7613360;rs7428430 |
| Indirect effect | rs11928368 | 3:85661265:G:T | 3 | 85661265 | 1.06393023241e-08 | 0.639 | 6 | CADM2 | intronic | 0 | 1 | rs11928368 |
| Indirect effect | rs62287547 | 4:10147342:C:T | 4 | 10147342 | 8.77033590083e-46 | 3.132 | 7 | RP11-480G3.1 | intergenic | 2063 | 1 | rs62287547 |
| Indirect effect | rs11096777 | 4:35568903:C:T | 4 | 35568903 | 1.20294143216e-09 | 1.036 | 7 | RNU6-573P | intergenic | 71278 | 2 | rs11096777;rs11729500 |
| Indirect effect | rs72678864 | 4:112422145:A:G | 4 | 112422145 | 4.33359151473e-10 | 21 | 7 | RP11-255I10.1 | intergenic | 50865 | 1 | rs72678864 |
| Indirect effect | rs6534338 | 4:123026869:C:T | 4 | 123026869 | 7.24718852556e-10 | 1.413 | 5 | RN7SL335P | intergenic | 17903 | 3 | rs6534338;rs45510500;rs11931332 |
| Indirect effect | rs57800857 | 4:140863365:A:C | 4 | 140863365 | 2.74804405397e-21 | 1.308 | 5 | MAML3 | intronic | 0 | 1 | rs57800857 |
| Indirect effect | rs7689452 | 4:147945733:A:G | 4 | 147945733 | 5.79081146947e-11 | 4.237 | 7 | RP11-292D4.3 | intergenic | 15436 | 3 | rs7689452;rs2118257;rs67970900 |
| Indirect effect | rs329120 | 5:133861756:C:T | 5 | 133861756 | 5.57853212469e-09 | 20.5 | 4 | JADE2 | intronic | 0 | 1 | rs329120 |
| Indirect effect | rs13179596 | 5:154779548:C:T | 5 | 154779548 | 4.32901614179e-08 | 0.173 | 7 | CTC-447K7.1 | intergenic | 1635 | 1 | rs13179596 |
| Indirect effect | rs185074 | 5:165284518:C:G | 5 | 165284518 | 4.98098604173e-08 | 0.08 | 6 | CTC-425K20.1 | intergenic | 47613 | 1 | rs185074 |
| Indirect effect | rs34502282 | 5:165699758:C:T | 5 | 165699758 | 1.21944108246e-09 | 1.196 | 6 | CTB-63M22.1 | intergenic | 109552 | 1 | rs34502282 |
| Indirect effect | rs1549212 | 5:166996722:C:T | 5 | 166996722 | 1.43027198501e-13 | 2.283 | 6 | TENM2 | intronic | 0 | 3 | rs1549212;rs7734360;rs1972645 |
| Indirect effect | rs11961437 | 6:112197053:A:C | 6 | 112197053 | 1.1791099616e-91 | 0.075 | 5 | FYN | intergenic | 2398 | 1 | rs11961437 |
| Indirect effect | rs6963853 | 7:1858725:A:G | 7 | 1858725 | 7.16090156822e-09 | 1.305 | 5 | MAD1L1 | intronic | 0 | 1 | rs6963853 |
| Indirect effect | rs7778443 | 7:32314690:C:T | 7 | 32314690 | 1.81373940578e-08 | 7.446 | 5 | PDE1C | intronic | 0 | 1 | rs7778443 |
| Indirect effect | rs114805382 | 7:108884701:A:C | 7 | 108884701 | 1.70101191686e-08 | 0.063 | 7 | AC002386.1 | intergenic | 77676 | 1 | rs114805382 |
| Indirect effect | rs12705966 | 7:114248851:A:G | 7 | 114248851 | 1.3877821081e-13 | 2.447 | 6 | FOXP2 | intronic | 0 | 4 | rs12705966;rs10953765;rs10228494;rs2079070 |
| Indirect effect | rs77236693 | 7:114620506:C:T | 7 | 114620506 | 4.45095104162e-09 | 11.39 | 5 | MDFIC | intronic | 0 | 1 | rs77236693 |
| Indirect effect | rs17137753 | 7:115030592:A:G | 7 | 115030592 | 2.96150298843e-14 | 3.971 | 7 | RP11-222O23.1 | intergenic | 112829 | 4 | rs17137753;rs6968125;rs6968380;rs12706030 |
| Indirect effect | rs10233018 | 7:117523709:A:G | 7 | 117523709 | 1.69960045202e-12 | 8.432 | 7 | AC007568.1 | ncRNA_exonic | 0 | 5 | rs10233018;rs34729439;rs10230941;rs8180706;rs7794364 |
| Indirect effect | rs11761835 | 7:118014841:C:T | 7 | 118014841 | 1.2064182071e-09 | 0.865 | 7 | AC006926.1 | intergenic | 87712 | 1 | rs11761835 |
| Indirect effect | rs73227362 | 8:27415350:A:C | 8 | 27415350 | 1.27520324854e-13 | 2.88 | 5 | GULOP | intergenic | 2441 | 7 | rs73227362;rs4732724;rs7831810;rs6558008;rs7012010;rs11780471;rs56223946 |
| Indirect effect | rs74779020 | 8:43221828:A:G | 8 | 43221828 | 1.71392179973e-81 | 1.589 | 7 | AC022616.1 | downstream | 0 | 1 | rs74779020 |
| Indirect effect | rs17643634 | 8:91650818:C:T | 8 | 91650818 | 3.36130267805e-08 | 3.176 | 7 | TMEM64 | intronic | 0 | 1 | rs17643634 |
| Indirect effect | rs2740792 | 8:91997679:A:G | 8 | 91997679 | 7.0728087816e-09 | 5.143 | NA | C8orf88 | upstream | 0 | 1 | rs2740792 |
| Indirect effect | rs1402940 | 8:114116686:G:T | 8 | 114116686 | 3.29650255068e-08 | 1.194 | 6 | CSMD3 | intronic | 0 | 1 | rs1402940 |
| Indirect effect | rs7020263 | 9:1715680:A:G | 9 | 1715680 | 3.68913005414e-38 | 2.539 | 7 | RP11-443B9.1 | intergenic | 262104 | 1 | rs7020263 |
| Indirect effect | rs4543592 | 9:3014254:C:T | 9 | 3014254 | 6.15785894206e-09 | 13.82 | 7 | CARM1P1 | ncRNA_intronic | 0 | 1 | rs4543592 |
| Indirect effect | rs633533 | 9:127819881:C:T | 9 | 127819881 | 6.68047647485e-12 | 1.437 | 4 | SCAI | intronic | 0 | 7 | rs633533;rs10121756;rs359587;rs3104549;rs12346687;rs10441758;rs360209 |
| Indirect effect | rs2819625 | 9:128156648:A:G | 9 | 128156648 | 1.67586928207e-12 | 0.078 | 4 | RP11-184B22.2 | intergenic | 21603 | 10 | rs2819625;rs2900227;rs13296519;rs10441758;rs13290762;rs360209;rs10121756;rs359587;rs3104549;rs12346687 |
| Indirect effect | rs7040561 | 9:128528978:A:T | 9 | 128528978 | 4.11784156388e-08 | 21.3 | 5 | PBX3 | intronic | 0 | 4 | rs7040561;rs3104549;rs13296519;rs10441758 |
| Indirect effect | rs3025316 | 9:136459543:C:T | 9 | 136459543 | 1.19390378418e-23 | 2.588 | 4 | FAM163B | intergenic | 8224 | 6 | rs3025316;rs3025335;rs3025363;rs7872903;rs28675164;rs17150650 |
| Indirect effect | rs1029372 | 9:136478442:A:G | 9 | 136478442 | 4.004849914e-12 | 0.86 | 5 | LL09NC01-254D11.1 | ncRNA_exonic | 0 | 4 | rs1029372;rs3025363;rs7872903;rs3025335 |
| Indirect effect | rs3025382 | 9:136502321:A:G | 9 | 136502321 | 3.05792305272e-08 | 0.796 | 4 | DBH | intronic | 0 | 1 | rs3025382 |
| Indirect effect | rs2797851 | 9:136504580:A:G | 9 | 136504580 | 4.66896070594e-08 | 0.177 | 1f | DBH | intronic | 0 | 1 | rs2797851 |
| Indirect effect | rs11255908 | 10:8802912:G:T | 10 | 8802912 | 8.56997365724e-11 | 10.72 | 6 | RP11-575N15.1 | intergenic | 36661 | 2 | rs11255908;rs3928823 |
| Indirect effect | rs2588978 | 10:63559609:C:T | 10 | 63559609 | 1.78534899328e-10 | 5.209 | 6 | RP11-491H19.1 | ncRNA_intronic | 0 | 3 | rs2588978;rs2393788;rs2588959 |
| Indirect effect | rs3850699 | 10:104414221:A:G | 10 | 104414221 | 7.97783285828e-10 | 1.046 | NA | TRIM8 | intronic | 0 | 2 | rs3850699;rs12764388 |
| Indirect effect | rs11191530 | 10:104800044:C:T | 10 | 104800044 | 7.0286366851e-09 | 1.407 | 1f | CNNM2 | intronic | 0 | 2 | rs11191530;rs12764388 |
| Indirect effect | rs11596214 | 10:106453832:A:G | 10 | 106453832 | 3.16947047276e-13 | 3.038 | 7 | SORCS3 | intronic | 0 | 3 | rs11596214;rs12416372;rs12250380 |
| Indirect effect | rs2463126 | 10:122195629:A:G | 10 | 122195629 | 1.9813006226e-08 | 11.46 | 5 | RP11-572P18.2 | ncRNA_exonic | 0 | 1 | rs2463126 |
| Indirect effect | rs72848823 | 10:132454993:A:G | 10 | 132454993 | 9.40081831095e-09 | 0.312 | 5 | Y_RNA | intergenic | 151528 | 1 | rs72848823 |
| Indirect effect | rs214104 | 11:17314784:A:G | 11 | 17314784 | 1.16875964024e-10 | 2.102 | NA | NUCB2 | intronic | 0 | 3 | rs214104;rs12794156;rs214904 |
| Indirect effect | rs11030382 | 11:28604477:C:T | 11 | 28604477 | 1.1599808061e-12 | 0.871 | 5 | RP11-22P4.1 | intergenic | 55889 | 2 | rs11030382;rs4391802 |
| Indirect effect | rs7948789 | 11:112839532:A:G | 11 | 112839532 | 1.18110112957e-18 | 13.84 | 5 | NCAM1 | intronic | 0 | 6 | rs7948789;rs12574676;rs1836799;rs1245094;rs1447481;rs57715877 |
| Indirect effect | rs7125588 | 11:113436072:A:G | 11 | 113436072 | 1.40457456168e-08 | 1.64 | 7 | DRD2 | intergenic | 89961 | 1 | rs7125588 |
| Indirect effect | rs73314417 | 12:53129744:C:T | 12 | 53129744 | 4.41849687016e-17 | 1.282 | 5 | KRT125P | ncRNA_exonic | 0 | 1 | rs73314417 |
| Indirect effect | rs10149845 | 14:30177079:C:T | 14 | 30177079 | 2.17286545372e-09 | 0.691 | 7 | PRKD1 | intronic | 0 | 1 | rs10149845 |
| Indirect effect | rs1890942 | 14:69765644:A:G | 14 | 69765644 | 8.14738601263e-09 | 2.496 | 5 | GALNT16 | intronic | 0 | 2 | rs1890942;rs1464307 |
| Indirect effect | rs10134246 | 14:80356531:A:G | 14 | 80356531 | 1.16684406812e-10 | 2.626 | 7 | RP11-526N18.1 | intergenic | 2892 | 1 | rs10134246 |
| Indirect effect | rs7144406 | 14:98659847:A:G | 14 | 98659847 | 8.17398987053e-09 | 0.629 | 7 | RP11-61O1.1 | ncRNA_intronic | 0 | 1 | rs7144406 |
| Indirect effect | rs11160771 | 14:104363322:G:T | 14 | 104363322 | 1.28788940936e-08 | 7.929 | 4 | C14orf2 | intergenic | 15303 | 1 | rs11160771 |
| Indirect effect | rs34488670 | 15:47684936:C:T | 15 | 47684936 | 5.5367701963e-15 | 1.429 | 5 | CTD-2050N2.1 | ncRNA_intronic | 0 | 9 | rs34488670;rs281297;rs281323;rs11856940;rs1466781;rs7169340;rs535072;rs17289269;rs4624112 |
| Indirect effect | rs1435742 | 15:47935785:A:G | 15 | 47935785 | 3.10120597684e-09 | 6.051 | NA | SEMA6D | intronic | 0 | 4 | rs1435742;rs535072;rs17289269;rs281323 |
| Indirect effect | rs55655049 | 15:78042708:C:T | 15 | 78042708 | 1.41809833805e-08 | 18.98 | 5 | LINGO1 | intronic | 0 | 1 | rs55655049 |
| Indirect effect | rs75157919 | 15:79248080:A:G | 15 | 79248080 | 4.20977389212e-19 | 1.265 | 7 | RASGRF1 | intergenic | 4209 | 1 | rs75157919 |
| Indirect effect | rs4778745 | 15:80363413:A:G | 15 | 80363413 | 4.94493655408e-08 | 19.93 | 4 | ZFAND6 | intronic | 0 | 1 | rs4778745 |
| Indirect effect | rs8031550 | 15:80956407:A:G | 15 | 80956407 | 4.19889271074e-09 | 0.89 | 5 | RNU6-380P | downstream | 0 | 1 | rs8031550 |
| Indirect effect | rs8026678 | 15:91562005:A:G | 15 | 91562005 | 2.18287250365e-09 | 0.876 | 6 | VPS33B | intronic | 0 | 1 | rs8026678 |
| Indirect effect | rs2017500 | 15:99196112:A:G | 15 | 99196112 | 4.52874376956e-08 | 1.7 | 3a | IGF1R | intronic | 0 | 1 | rs2017500 |
| Indirect effect | rs58189590 | 16:85986059:A:G | 16 | 85986059 | 3.67134047535e-26 | 0.232 | 5 | RP11-542M13.3 | intergenic | 16230 | 1 | rs58189590 |
| Indirect effect | rs8069260 | 17:18677362:A:G | 17 | 18677362 | 1.86126315274e-22 | 3.401 | 3a | FBXW10 | intronic | 0 | 1 | rs8069260 |
| Indirect effect | rs112178027 | 17:27564013:C:T | 17 | 27564013 | 2.69649711586e-08 | 7.033 | 5 | CRYBA1 | intergenic | 9868 | 2 | rs112178027;rs60616188 |
| Indirect effect | rs12967855 | 18:35138245:A:G | 18 | 35138245 | 1.42884658815e-08 | 0.424 | 5 | CELF4 | intronic | 0 | 1 | rs12967855 |
| Indirect effect | rs17504372 | 18:50903050:C:T | 18 | 50903050 | 6.05842342494e-09 | 0.434 | 6 | DCC | intronic | 0 | 1 | rs17504372 |
| Indirect effect | rs1452787 | 18:53207207:A:G | 18 | 53207207 | 9.88978123956e-10 | 0.08 | 7 | TCF4 | intronic | 0 | 1 | rs1452787 |
| Indirect effect | rs71367544 | 18:77574374:C:T | 18 | 77574374 | 6.15403569198e-09 | 0.012 | 5 | RP11-154H12.3 | ncRNA_intronic | 0 | 1 | rs71367544 |
| Indirect effect | rs113050285 | 19:21232539:C:G | 19 | 21232539 | 2.6227275814e-18 | 3.555 | 5 | ZNF430 | intronic | 0 | 1 | rs113050285 |
| Indirect effect | rs11878604 | 19:41333284:C:T | 19 | 41333284 | 1.86780294948e-08 | 5.035 | 2b | CTC-490E21.12 | intronic | 0 | 2 | rs11878604;rs12459249 |
| Indirect effect | rs56113850 | 19:41353107:C:T | 19 | 41353107 | 2.26196109857e-27 | 1.127 | 5 | CYP2A6 | intronic | 0 | 7 | rs56113850;rs7250713;rs12610432;rs10425738;rs2644891;rs7255471;rs12459249 |
| Indirect effect | rs184589612 | 19:41412192:C:T | 19 | 41412192 | 2.67676182486e-09 | 0.41 | 5 | CTC-490E21.13 | intergenic | 2185 | 2 | rs184589612;rs112292570 |

Table S4. CADD score

| **uniqID** | **GWAS** | **CADD** | **symbol** | **annot** | **dist** | **exonic_func** | **chr** | **pos** |
| --- | --- | --- | --- | --- | --- | --- | --- | --- |
| 4:123179900:C:T | Indirect effect | 29.1 | KIAA1109 | exonic | 0 | nonsynonymous SNV | 4 | 123179900 |
| 17:18653188:G:T | Indirect effect | 24.3 | FBXW10 | exonic | 0 | nonsynonymous SNV | 17 | 18653188 |
| 10:104474046:C:CCCT | Indirect effect | 22.7 | ARL3 | UTR5 | 0 | NA | 10 | 104474046 |
| 10:104934709:C:T | Indirect effect | 22.7 | NT5C2 | exonic | 0 | nonsynonymous SNV | 10 | 104934709 |
| 9:127818276:C:T | Indirect effect | 22.1 | SCAI | exonic | 0 | nonsynonymous SNV | 9 | 127818276 |
| 9:128141809:A:G | Indirect effect | 22.1 | RP11-184B22.2 | intergenic | 6764 | NA | 9 | 128141809 |
| 15:91543761:C:T | Indirect effect | 22 | VPS33B | exonic | 0 | nonsynonymous SNV | 15 | 91543761 |
| 9:128655115:G:T | Indirect effect | 22 | PBX3 | intronic | 0 | NA | 9 | 128655115 |
| 2:105954140:A:G | Indirect effect | 21.7 | C2orf49 | exonic | 0 | synonymous SNV | 2 | 105954140 |
| 7:114940159:A:G | Indirect effect | 21.6 | AC068610.5 | intergenic | 68750 | NA | 7 | 114940159 |
| 7:115027979:A:T | Indirect effect | 21.6 | RP11-222O23.1 | intergenic | 115442 | NA | 7 | 115027979 |
| 7:114940147:C:T | Indirect effect | 21.4 | AC068610.5 | intergenic | 68738 | NA | 7 | 114940147 |
| 9:127909622:C:G | Indirect effect | 21.3 | PPP6C | UTR3 | 0 | NA | 9 | 127909622 |
| 9:128528978:A:T | Indirect effect | 21.3 | PBX3 | intronic | 0 | NA | 9 | 128528978 |
| 11:17351683:C:G | Indirect effect | 21.1 | NUCB2 | exonic | 0 | nonsynonymous SNV | 11 | 17351683 |
| 15:47657135:A:G | Indirect effect | 21.1 | CTD-2050N2.1 | ncRNA_intronic | 0 | NA | 15 | 47657135 |
| 2:145951277:C:T | Indirect effect | 21.1 | TEX41 | intergenic | 11061 | NA | 2 | 145951277 |
| 5:167027636:A:G | Indirect effect | 21.1 | TENM2 | intronic | 0 | NA | 5 | 167027636 |
| 9:128514608:C:T | Indirect effect | 21.1 | PBX3 | intronic | 0 | NA | 9 | 128514608 |
| 15:47566333:A:G | Indirect effect | 21 | SEMA6D | intronic | 0 | NA | 15 | 47566333 |
| 4:112422145:A:G | Indirect effect | 21 | RP11-255I10.1 | intergenic | 50865 | NA | 4 | 112422145 |
| 11:113438092:A:C | Indirect effect | 20.9 | DRD2 | intergenic | 91981 | NA | 11 | 113438092 |
| 15:47641602:C:G | Indirect effect | 20.9 | SEMA6D | intronic | 0 | NA | 15 | 47641602 |
| 2:146138048:C:T | Indirect effect | 20.9 | RPL6P5 | intergenic | 42423 | NA | 2 | 146138048 |
| 9:128592201:A:G | Indirect effect | 20.9 | PBX3 | intronic | 0 | NA | 9 | 128592201 |
| 5:154894169:A:G | Indirect effect | 20.8 | CTB-95D12.1 | intergenic | 21011 | NA | 5 | 154894169 |
| 7:114303833:A:G | Indirect effect | 20.8 | FOXP2 | intronic | 0 | NA | 7 | 114303833 |
| 9:128406988:C:G | Indirect effect | 20.8 | MAPKAP1 | intronic | 0 | NA | 9 | 128406988 |
| 9:128303942:C:G | Indirect effect | 20.7 | MAPKAP1 | intronic | 0 | NA | 9 | 128303942 |
| 9:128363447:G:GGTTATA | Indirect effect | 20.7 | MAPKAP1 | intronic | 0 | NA | 9 | 128363447 |
| 10:106632655:C:T | Indirect effect | 20.6 | SORCS3 | intronic | 0 | NA | 10 | 106632655 |
| 5:133861756:C:T | Indirect effect | 20.5 | JADE2 | intronic | 0 | NA | 5 | 133861756 |
| 10:106467853:A:G | Indirect effect | 20.4 | SORCS3 | intronic | 0 | NA | 10 | 106467853 |
| 9:128521473:C:T | Indirect effect | 20.4 | PBX3 | intronic | 0 | NA | 9 | 128521473 |
| 9:128442229:A:G | Indirect effect | 20.3 | MAPKAP1 | intronic | 0 | NA | 9 | 128442229 |
| 17:27413115:C:T | Indirect effect | 20.2 | MYO18A | intronic | 0 | NA | 17 | 27413115 |
| 2:203765756:C:T | Indirect effect | 20.2 | WDR12 | exonic | 0 | nonsynonymous SNV | 2 | 203765756 |
| 2:146261295:C:T | Indirect effect | 20.1 | AC092484.1 | intergenic | 65567 | NA | 2 | 146261295 |
| 9:128640702:C:T | Indirect effect | 20.1 | PBX3 | intronic | 0 | NA | 9 | 128640702 |
| 4:140856533:A:G | Indirect effect | 19.95 | MAML3 | intronic | 0 | NA | 4 | 140856533 |
| 15:80363413:A:G | Indirect effect | 19.93 | ZFAND6 | intronic | 0 | NA | 15 | 80363413 |
| 7:115069789:A:G | Indirect effect | 19.92 | RP11-222O23.1 | intergenic | 73632 | NA | 7 | 115069789 |
| 5:167039800:A:G | Indirect effect | 19.91 | TENM2 | intronic | 0 | NA | 5 | 167039800 |
| 14:98629253:C:T | Indirect effect | 19.88 | RP11-61O1.1 | ncRNA_intronic | 0 | NA | 14 | 98629253 |
| 2:146355456:C:T | Indirect effect | 19.72 | AC079163.1 | intergenic | 3019 | NA | 2 | 146355456 |
| 18:35145122:C:T | Indirect effect | 19.67 | CELF4 | intronic | 0 | NA | 18 | 35145122 |
| 10:106534351:C:T | Indirect effect | 19.55 | SORCS3 | intronic | 0 | NA | 10 | 106534351 |
| 7:114296102:C:T | Indirect effect | 19.46 | FOXP2 | intronic | 0 | NA | 7 | 114296102 |
| 9:128524269:A:G | Indirect effect | 19.44 | PBX3 | intronic | 0 | NA | 9 | 128524269 |
| 9:128655271:A:T | Indirect effect | 19.41 | PBX3 | intronic | 0 | NA | 9 | 128655271 |
| 5:167001332:A:G | Indirect effect | 19.37 | TENM2 | intronic | 0 | NA | 5 | 167001332 |
| 9:128525034:A:G | Indirect effect | 19.35 | PBX3 | intronic | 0 | NA | 9 | 128525034 |
| 15:47641899:A:G | Indirect effect | 19.29 | SEMA6D | intronic | 0 | NA | 15 | 47641899 |
| 7:114957212:T:TGAG | Indirect effect | 19.26 | AC068610.5 | intergenic | 85803 | NA | 7 | 114957212 |
| 2:186035745:C:T | Indirect effect | 19.24 | ZNF804A | intergenic | 231526 | NA | 2 | 186035745 |
| 2:145955643:A:C | Indirect effect | 19.21 | TEX41 | intergenic | 15427 | NA | 2 | 145955643 |
| 2:146168208:G:T | Indirect effect | 19.12 | RPL6P5 | intergenic | 72583 | NA | 2 | 146168208 |
| 5:154833501:A:T | Indirect effect | 19.1 | CTB-95D12.1 | intergenic | 37395 | NA | 5 | 154833501 |
| 10:106521003:C:T | Indirect effect | 19.08 | SORCS3 | intronic | 0 | NA | 10 | 106521003 |
| 7:114304969:C:T | Indirect effect | 19.08 | FOXP2 | UTR3 | 0 | NA | 7 | 114304969 |
| 14:30073056:C:T | Indirect effect | 19.07 | PRKD1 | intronic | 0 | NA | 14 | 30073056 |
| 15:47676716:C:T | Indirect effect | 19.02 | CTD-2050N2.1 | ncRNA_intronic | 0 | NA | 15 | 47676716 |
| 9:128646863:A:T | Indirect effect | 19 | PBX3 | intronic | 0 | NA | 9 | 128646863 |
| 15:78042708:C:T | Indirect effect | 18.98 | LINGO1 | intronic | 0 | NA | 15 | 78042708 |
| 1:50572096:A:C | Indirect effect | 18.94 | ELAVL4 | intronic | 0 | NA | 1 | 50572096 |
| 5:166993972:A:G | Indirect effect | 18.94 | TENM2 | intronic | 0 | NA | 5 | 166993972 |
| 9:128225561:C:T | Indirect effect | 18.85 | MAPKAP1 | intronic | 0 | NA | 9 | 128225561 |
| 5:167035581:A:G | Indirect effect | 18.76 | TENM2 | intronic | 0 | NA | 5 | 167035581 |
| 10:63557648:C:T | Indirect effect | 18.75 | RP11-491H19.1 | ncRNA_intronic | 0 | NA | 10 | 63557648 |
| 11:28688407:A:G | Indirect effect | 18.74 | RP11-960D24.1 | intergenic | 12323 | NA | 11 | 28688407 |
| 9:128724683:A:C | Indirect effect | 18.7 | PBX3 | intronic | 0 | NA | 9 | 128724683 |
| 3:50176259:A:AG | Indirect effect | 18.58 | RP11-493K19.3 | ncRNA_intronic | 0 | NA | 3 | 50176259 |
| 10:63600048:C:T | Indirect effect | 18.57 | RP11-491H19.1 | intergenic | 19044 | NA | 10 | 63600048 |
| 18:50749935:A:T | Indirect effect | 18.56 | DCC | intronic | 0 | NA | 18 | 50749935 |
| 4:35517877:C:T | Indirect effect | 18.54 | RNU6-573P | intergenic | 20252 | NA | 4 | 35517877 |
| 3:49915506:A:T | Indirect effect | 18.51 | ACTBP13 | upstream | 0 | NA | 3 | 49915506 |
| 2:45121466:A:G | Indirect effect | 18.48 | RP11-89K21.1 | intergenic | 25866 | NA | 2 | 45121466 |
| 3:50153356:C:T | Indirect effect | 18.47 | RBM5 | exonic | 0 | synonymous SNV | 3 | 50153356 |
| 8:114186003:C:T | Indirect effect | 18.45 | CSMD3 | exonic | 0 | nonsynonymous SNV | 8 | 114186003 |
| 15:47641933:A:AT | Indirect effect | 18.28 | SEMA6D | intronic | 0 | NA | 15 | 47641933 |
| 2:61765074:A:G | Indirect effect | 18.28 | XPO1 | UTR5 | 0 | NA | 2 | 61765074 |
| 1:73917111:A:C | Indirect effect | 18.22 | RP4-598G3.1 | intergenic | 96177 | NA | 1 | 73917111 |
| 7:114174361:T:TA | Indirect effect | 18.18 | FOXP2 | intronic | 0 | NA | 7 | 114174361 |
| 1:44007366:G:GGACAGTTCTTCC | Indirect effect | 18.13 | PTPRF | intronic | 0 | NA | 1 | 44007366 |
| 9:128490151:C:T | Indirect effect | 18.13 | RP11-423C15.3 | intergenic | 15882 | NA | 9 | 128490151 |
| 7:117603820:C:G | Indirect effect | 18.12 | AC003084.2 | intergenic | 35092 | NA | 7 | 117603820 |
| 2:45165045:A:C | Indirect effect | 18.09 | RP11-89K21.1 | ncRNA_intronic | 0 | NA | 2 | 45165045 |
| 2:161283398:C:T | Indirect effect | 18.04 | RBMS1 | intronic | 0 | NA | 2 | 161283398 |
| 5:167039610:C:T | Indirect effect | 18 | TENM2 | intronic | 0 | NA | 5 | 167039610 |
| 1:44013355:A:G | Indirect effect | 17.97 | PTPRF | intronic | 0 | NA | 1 | 44013355 |
| 4:147966066:A:G | Indirect effect | 17.97 | RP11-292D4.3 | intergenic | 35769 | NA | 4 | 147966066 |
| 4:35556492:C:G | Indirect effect | 17.97 | RNU6-573P | intergenic | 58867 | NA | 4 | 35556492 |
| 9:128225199:A:G | Indirect effect | 17.91 | MAPKAP1 | intronic | 0 | NA | 9 | 128225199 |
| 4:35556646:A:C | Indirect effect | 17.81 | RNU6-573P | intergenic | 59021 | NA | 4 | 35556646 |
| 5:154848706:A:G | Indirect effect | 17.78 | CTB-95D12.1 | intergenic | 22190 | NA | 5 | 154848706 |
| 3:50139142:A:G | Indirect effect | 17.77 | RBM5 | UTR3 | 0 | NA | 3 | 50139142 |
| 9:128269511:C:T | Indirect effect | 17.76 | MAPKAP1 | intronic | 0 | NA | 9 | 128269511 |
| 15:47876710:C:T | Indirect effect | 17.68 | SEMA6D | intronic | 0 | NA | 15 | 47876710 |
| 2:45154908:A:G | Indirect effect | 17.67 | RP11-89K21.1 | ncRNA_intronic | 0 | NA | 2 | 45154908 |
| 9:128721828:C:G | Indirect effect | 17.66 | PBX3 | intronic | 0 | NA | 9 | 128721828 |
| 9:128139084:C:T | Indirect effect | 17.62 | RP11-184B22.2 | intergenic | 4039 | NA | 9 | 128139084 |
| 2:32713706:A:T | Indirect effect | 17.61 | BIRC6 | exonic | 0 | nonsynonymous SNV | 2 | 32713706 |
| 3:49898273:A:G | Indirect effect | 17.61 | CAMKV | exonic | 0 | synonymous SNV | 3 | 49898273 |
| 15:47660022:A:T | Indirect effect | 17.58 | CTD-2050N2.1 | ncRNA_intronic | 0 | NA | 15 | 47660022 |
| 1:50579237:A:C | Indirect effect | 17.57 | ELAVL4 | intronic | 0 | NA | 1 | 50579237 |
| 18:35159124:C:T | Indirect effect | 17.54 | CELF4 | intergenic | 13124 | NA | 18 | 35159124 |
| 2:22439514:A:C | Indirect effect | 17.54 | AC068490.2 | ncRNA_intronic | 0 | NA | 2 | 22439514 |
| 15:47677087:A:G | Indirect effect | 17.53 | CTD-2050N2.1 | ncRNA_intronic | 0 | NA | 15 | 47677087 |
| 3:49911155:A:G | Indirect effect | 17.49 | ACTBP13 | ncRNA_exonic | 0 | NA | 3 | 49911155 |
| 2:203777226:C:T | Indirect effect | 17.48 | CARF | UTR5 | 0 | NA | 2 | 203777226 |
| 10:106469813:A:G | Indirect effect | 17.46 | SORCS3 | intronic | 0 | NA | 10 | 106469813 |
| 15:47493363:G:T | Indirect effect | 17.43 | SEMA6D | intronic | 0 | NA | 15 | 47493363 |
| 1:44018604:G:T | Indirect effect | 17.42 | PTPRF | intronic | 0 | NA | 1 | 44018604 |
| 8:114162023:A:G | Indirect effect | 17.4 | CSMD3 | intronic | 0 | NA | 8 | 114162023 |
| 4:140883618:C:T | Indirect effect | 17.39 | MAML3 | intronic | 0 | NA | 4 | 140883618 |
| 7:114072443:A:G | Indirect effect | 17.34 | FOXP2 | intronic | 0 | NA | 7 | 114072443 |
| 5:165747886:A:G | Indirect effect | 17.31 | CTB-63M22.1 | intergenic | 61424 | NA | 5 | 165747886 |
| 9:128726204:C:CT | Indirect effect | 17.31 | PBX3 | intronic | 0 | NA | 9 | 128726204 |
| 10:106631330:G:T | Indirect effect | 17.3 | SORCS3 | intronic | 0 | NA | 10 | 106631330 |
| 10:63596035:C:G | Indirect effect | 17.29 | RP11-491H19.1 | intergenic | 15031 | NA | 10 | 63596035 |
| 5:133861663:C:T | Indirect effect | 17.26 | JADE2 | UTR5 | 0 | NA | 5 | 133861663 |
| 15:47683856:A:AT | Indirect effect | 17.2 | CTD-2050N2.1 | ncRNA_intronic | 0 | NA | 15 | 47683856 |
| 11:113438068:A:G | Indirect effect | 17.19 | DRD2 | intergenic | 91957 | NA | 11 | 113438068 |
| 2:45167570:G:T | Indirect effect | 17.15 | SIX3-AS1 | ncRNA_exonic | 0 | NA | 2 | 45167570 |
| 5:167003336:A:AT | Indirect effect | 17.12 | NA | NA | NA | NA | NA | NA |
| 5:167003336:A:ATT | Indirect effect | 17.09 | TENM2 | intronic | 0 | NA | 5 | 167003336 |
| 9:136474577:C:CGG | Indirect effect | 17.09 | LL09NC01-254D11.1 | intergenic | 2341 | NA | 9 | 136474577 |
| 10:104429746:C:G | Indirect effect | 17 | ARL3 | intergenic | 3742 | NA | 10 | 104429746 |
| 11:28625357:A:T | Indirect effect | 16.96 | RP11-960D24.1 | intergenic | 75373 | NA | 11 | 28625357 |
| 9:136405965:A:C | Indirect effect | 16.96 | ADAMTSL2 | intronic | 0 | NA | 9 | 136405965 |
| 18:50949402:A:C | Indirect effect | 16.95 | DCC | intronic | 0 | NA | 18 | 50949402 |
| 7:114056495:A:AAATTTCAT | Indirect effect | 16.95 | AC073626.2 | ncRNA_intronic | 0 | NA | 7 | 114056495 |
| 10:63561316:A:G | Indirect effect | 16.92 | RP11-491H19.1 | ncRNA_intronic | 0 | NA | 10 | 63561316 |
| 15:47676579:A:T | Indirect effect | 16.9 | CTD-2050N2.1 | ncRNA_intronic | 0 | NA | 15 | 47676579 |
| 17:27393310:A:G | Indirect effect | 16.9 | MYO18A | intergenic | 7218 | NA | 17 | 27393310 |
| 5:167003368:A:G | Indirect effect | 16.87 | TENM2 | intronic | 0 | NA | 5 | 167003368 |
| 7:114069536:C:G | Indirect effect | 16.86 | FOXP2 | intronic | 0 | NA | 7 | 114069536 |
| 2:145885908:A:AC | Indirect effect | 16.85 | TEX41 | ncRNA_intronic | 0 | NA | 2 | 145885908 |
| 9:3018365:A:C | Indirect effect | 16.85 | CARM1P1 | ncRNA_intronic | 0 | NA | 9 | 3018365 |
| 10:106528466:A:G | Indirect effect | 16.83 | SORCS3 | intronic | 0 | NA | 10 | 106528466 |
| 1:49511424:A:G | Indirect effect | 16.78 | AGBL4 | exonic | 0 | synonymous SNV | 1 | 49511424 |
| 7:114056055:C:G | Indirect effect | 16.74 | AC073626.2 | ncRNA_intronic | 0 | NA | 7 | 114056055 |
| 10:106544636:A:T | Indirect effect | 16.63 | SORCS3 | intronic | 0 | NA | 10 | 106544636 |
| 9:127913764:A:G | Indirect effect | 16.55 | PRPS1P2 | ncRNA_exonic | 0 | NA | 9 | 127913764 |
| 18:50906636:C:T | Indirect effect | 16.52 | DCC | intronic | 0 | NA | 18 | 50906636 |
| 9:128717131:G:T | Indirect effect | 16.47 | PBX3 | intronic | 0 | NA | 9 | 128717131 |
| 7:118007961:A:G | Indirect effect | 16.43 | AC006926.1 | intergenic | 94592 | NA | 7 | 118007961 |
| 7:32333921:A:C | Indirect effect | 16.42 | PDE1C | intronic | 0 | NA | 7 | 32333921 |
| 4:35552282:A:G | Indirect effect | 16.4 | RNU6-573P | intergenic | 54657 | NA | 4 | 35552282 |
| 9:127910498:G:T | Indirect effect | 16.35 | PPP6C | UTR3 | 0 | NA | 9 | 127910498 |
| 2:203848402:A:G | Indirect effect | 16.28 | CARF | UTR3 | 0 | NA | 2 | 203848402 |
| 15:78964362:C:T | Direct effect | 16.26 | RP11-160C18.2 | ncRNA_intronic | 0 | NA | 15 | 78964362 |
| 15:47644528:A:G | Indirect effect | 16.25 | SEMA6D | intronic | 0 | NA | 15 | 47644528 |
| 3:49449638:A:G | Indirect effect | 16.24 | RHOA | upstream | 0 | NA | 3 | 49449638 |
| 10:104795134:C:T | Indirect effect | 16.23 | CNNM2 | intronic | 0 | NA | 10 | 104795134 |
| 7:114297820:A:G | Indirect effect | 16.19 | FOXP2 | intronic | 0 | NA | 7 | 114297820 |
| 9:128003788:G:GC | Indirect effect | 16.18 | HSPA5 | upstream | 0 | NA | 9 | 128003788 |
| 9:127913974:A:T | Indirect effect | 16.17 | PPP6C | intronic | 0 | NA | 9 | 127913974 |
| 9:128367886:C:T | Indirect effect | 16.17 | MAPKAP1 | intronic | 0 | NA | 9 | 128367886 |
| 7:114171145:A:G | Indirect effect | 16.15 | FOXP2 | intronic | 0 | NA | 7 | 114171145 |
| 9:128689740:A:T | Indirect effect | 16.1 | PBX3 | intronic | 0 | NA | 9 | 128689740 |
| 2:31799509:C:T | Indirect effect | 16.06 | SRD5A2 | ncRNA_intronic | 0 | NA | 2 | 31799509 |
| 15:47916734:G:GA | Indirect effect | 16.05 | SEMA6D | intronic | 0 | NA | 15 | 47916734 |
| 7:117506810:A:T | Indirect effect | 16.03 | CTTNBP2 | intronic | 0 | NA | 7 | 117506810 |
| 14:30155193:C:T | Indirect effect | 16 | PRKD1 | intronic | 0 | NA | 14 | 30155193 |
| 14:30093013:C:T | Indirect effect | 15.98 | PRKD1 | intronic | 0 | NA | 14 | 30093013 |
| 9:128515046:A:C | Indirect effect | 15.98 | PBX3 | intronic | 0 | NA | 9 | 128515046 |
| 2:203739856:C:G | Indirect effect | 15.97 | WDR12 | UTR3 | 0 | NA | 2 | 203739856 |
| 15:47933163:C:T | Indirect effect | 15.95 | SEMA6D | intronic | 0 | NA | 15 | 47933163 |
| 2:145952930:T:TGAA | Indirect effect | 15.95 | TEX41 | intergenic | 12714 | NA | 2 | 145952930 |
| 7:117514840:G:T | Indirect effect | 15.94 | CTTNBP2 | upstream | 0 | NA | 7 | 117514840 |
| 7:114297180:A:G | Indirect effect | 15.93 | FOXP2 | intronic | 0 | NA | 7 | 114297180 |
| 7:114301107:C:CT | Indirect effect | 15.88 | FOXP2 | intronic | 0 | NA | 7 | 114301107 |
| 1:44021456:C:G | Indirect effect | 15.84 | PTPRF | intronic | 0 | NA | 1 | 44021456 |
| 1:50537618:C:T | Indirect effect | 15.84 | ELAVL4 | intronic | 0 | NA | 1 | 50537618 |
| 17:1684605:G:T | Direct effect | 15.83 | SMYD4 | exonic | 0 | nonsynonymous SNV | 17 | 1684605 |
| 5:167043098:A:G | Indirect effect | 15.81 | TENM2 | intronic | 0 | NA | 5 | 167043098 |
| 9:127909069:C:T | Indirect effect | 15.81 | PPP6C | UTR3 | 0 | NA | 9 | 127909069 |
| 9:127910777:A:AAAG | Indirect effect | 15.81 | PPP6C | UTR3 | 0 | NA | 9 | 127910777 |
| 7:118190093:A:G | Indirect effect | 15.8 | AC006926.1 | intergenic | 87445 | NA | 7 | 118190093 |
| 3:49054692:C:T | Indirect effect | 15.78 | DALRD3 | exonic | 0 | nonsynonymous SNV | 3 | 49054692 |
| 9:128407298:A:G | Indirect effect | 15.77 | MAPKAP1 | intronic | 0 | NA | 9 | 128407298 |
| 4:140877659:A:G | Indirect effect | 15.74 | MAML3 | intronic | 0 | NA | 4 | 140877659 |
| 9:127911031:A:G | Indirect effect | 15.74 | PPP6C | UTR3 | 0 | NA | 9 | 127911031 |
| 1:73852090:C:T | Indirect effect | 15.7 | RP4-598G3.1 | intergenic | 31156 | NA | 1 | 73852090 |
| 7:114303189:A:G | Indirect effect | 15.68 | FOXP2 | intronic | 0 | NA | 7 | 114303189 |
| 15:47641522:C:T | Indirect effect | 15.66 | SEMA6D | intronic | 0 | NA | 15 | 47641522 |
| 2:22441768:C:CT | Indirect effect | 15.66 | AC068490.2 | ncRNA_intronic | 0 | NA | 2 | 22441768 |
| 9:128723228:T:TG | Indirect effect | 15.63 | PBX3 | intronic | 0 | NA | 9 | 128723228 |
| 2:203846817:A:T | Indirect effect | 15.61 | CARF | exonic | 0 | nonsynonymous SNV | 2 | 203846817 |
| 9:127951863:A:G | Indirect effect | 15.58 | PPP6C | exonic | 0 | synonymous SNV | 9 | 127951863 |
| 14:30137407:C:T | Indirect effect | 15.57 | PRKD1 | intronic | 0 | NA | 14 | 30137407 |
| 1:44068275:A:G | Indirect effect | 15.53 | PTPRF | intronic | 0 | NA | 1 | 44068275 |
| 7:117652571:A:C | Indirect effect | 15.51 | AC003084.2 | intergenic | 8472 | NA | 7 | 117652571 |
| 8:91852827:C:T | Indirect effect | 15.43 | RP11-662G23.1 | ncRNA_intronic | 0 | NA | 8 | 91852827 |
| 7:115116526:C:G | Indirect effect | 15.4 | RP11-222O23.1 | intergenic | 26895 | NA | 7 | 115116526 |
| 4:147685521:G:T | Indirect effect | 15.39 | TTC29 | intronic | 0 | NA | 4 | 147685521 |
| 4:140867514:A:G | Indirect effect | 15.37 | MAML3 | intronic | 0 | NA | 4 | 140867514 |
| 1:44011737:A:G | Indirect effect | 15.32 | PTPRF | intronic | 0 | NA | 1 | 44011737 |
| 2:186031380:C:T | Indirect effect | 15.31 | ZNF804A | intergenic | 227161 | NA | 2 | 186031380 |
| 10:106536536:C:G | Indirect effect | 15.29 | SORCS3 | intronic | 0 | NA | 10 | 106536536 |
| 5:165686220:C:G | Indirect effect | 15.29 | AC122720.1 | intergenic | 99695 | NA | 5 | 165686220 |
| 15:78911181:C:T | Direct effect | 15.25 | CHRNA3 | exonic | 0 | synonymous SNV | 15 | 78911181 |
| 15:79112837:C:T | Direct effect | 15.2 | MORF4L1 | intronic | 0 | NA | 15 | 79112837 |
| 10:106611259:A:G | Indirect effect | 15.17 | SORCS3 | intronic | 0 | NA | 10 | 106611259 |
| 2:203739970:G:T | Indirect effect | 15.15 | WDR12 | UTR3 | 0 | NA | 2 | 203739970 |
| 3:49210732:A:C | Indirect effect | 15.13 | KLHDC8B | intronic | 0 | NA | 3 | 49210732 |
| 17:1665330:A:C | Direct effect | 15.12 | SERPINF1 | UTR5 | 0 | NA | 17 | 1665330 |
| 18:53270902:C:G | Indirect effect | 15.12 | TCF4 | intronic | 0 | NA | 18 | 53270902 |
| 9:127913970:A:AGT | Indirect effect | 15.12 | PPP6C | intronic | 0 | NA | 9 | 127913970 |
| 15:47515774:G:GTTGTCATCTTA | Indirect effect | 15.1 | SEMA6D | intronic | 0 | NA | 15 | 47515774 |
| 15:47923520:G:T | Indirect effect | 15.09 | SEMA6D | intronic | 0 | NA | 15 | 47923520 |
| 4:35435741:A:G | Indirect effect | 15.07 | SEC63P2 | intergenic | 53693 | NA | 4 | 35435741 |
| 4:35567847:A:C | Indirect effect | 15.07 | RNU6-573P | intergenic | 70222 | NA | 4 | 35567847 |
| 5:154865288:A:G | Indirect effect | 15.05 | CTB-95D12.1 | intergenic | 5608 | NA | 5 | 154865288 |
| 7:117548297:G:T | Indirect effect | 15.05 | AC007568.1 | intergenic | 24568 | NA | 7 | 117548297 |
| 5:167003569:G:T | Indirect effect | 15.02 | TENM2 | intronic | 0 | NA | 5 | 167003569 |
| 9:128662203:A:ATTTTCTTGATATTAGC | Indirect effect | 15.01 | PBX3 | intronic | 0 | NA | 9 | 128662203 |
| 11:28642381:C:T | Indirect effect | 15 | RP11-960D24.1 | intergenic | 58349 | NA | 11 | 28642381 |
| 15:47644573:C:T | Indirect effect | 14.99 | SEMA6D | intronic | 0 | NA | 15 | 47644573 |
| 1:44036085:A:G | Indirect effect | 14.99 | PTPRF | intronic | 0 | NA | 1 | 44036085 |
| 15:78882925:A:G | Direct effect | 14.97 | CHRNA5 | exonic | 0 | nonsynonymous SNV | 15 | 78882925 |
| 15:47834003:A:T | Indirect effect | 14.95 | SEMA6D | intronic | 0 | NA | 15 | 47834003 |
| 1:73851752:A:G | Indirect effect | 14.93 | RP4-598G3.1 | intergenic | 30818 | NA | 1 | 73851752 |
| 10:8802838:C:T | Indirect effect | 14.92 | RP11-575N15.1 | intergenic | 36587 | NA | 10 | 8802838 |
| 9:128365953:A:G | Indirect effect | 14.92 | MAPKAP1 | intronic | 0 | NA | 9 | 128365953 |
| 7:114129137:A:G | Indirect effect | 14.87 | FOXP2 | intronic | 0 | NA | 7 | 114129137 |
| 9:128025962:A:ACAGCACTCCATCTGTAGGTATGTCTGT | Indirect effect | 14.87 | GAPVD1 | intronic | 0 | NA | 9 | 128025962 |
| 9:128072273:C:G | Indirect effect | 14.87 | GAPVD1 | exonic | 0 | NA | 9 | 128072273 |
| 15:47856831:C:T | Indirect effect | 14.86 | SEMA6D | intronic | 0 | NA | 15 | 47856831 |
| 7:117801067:A:C | Indirect effect | 14.85 | NAA38 | intergenic | 23019 | NA | 7 | 117801067 |
| 2:161335574:C:T | Indirect effect | 14.79 | RBMS1 | intronic | 0 | NA | 2 | 161335574 |
| 8:91913568:C:T | Indirect effect | 14.77 | NECAB1 | intronic | 0 | NA | 8 | 91913568 |
| 1:73773048:C:T | Indirect effect | 14.76 | RP4-598G3.1 | ncRNA_exonic | 0 | NA | 1 | 73773048 |
| 3:85654340:C:G | Indirect effect | 14.76 | CADM2 | intronic | 0 | NA | 3 | 85654340 |
| 11:17259967:A:G | Indirect effect | 14.73 | NUCB2 | intronic | 0 | NA | 11 | 17259967 |
| 7:117506858:C:G | Indirect effect | 14.73 | CTTNBP2 | intronic | 0 | NA | 7 | 117506858 |
| 11:112903595:A:G | Indirect effect | 14.71 | NCAM1 | intronic | 0 | NA | 11 | 112903595 |
| 7:114294405:C:G | Indirect effect | 14.71 | FOXP2 | intronic | 0 | NA | 7 | 114294405 |
| 9:127913973:C:G | Indirect effect | 14.7 | PPP6C | intronic | 0 | NA | 9 | 127913973 |
| 17:1683317:C:G | Direct effect | 14.68 | SMYD4 | UTR3 | 0 | NA | 17 | 1683317 |
| 17:27588980:C:CAG | Indirect effect | 14.68 | NUFIP2 | UTR3 | 0 | NA | 17 | 27588980 |
| 2:161290337:A:C | Indirect effect | 14.67 | RBMS1 | intronic | 0 | NA | 2 | 161290337 |
| 11:112852192:C:T | Indirect effect | 14.65 | NCAM1 | intronic | 0 | NA | 11 | 112852192 |
| 7:117972595:A:G | Indirect effect | 14.64 | ANKRD7 | intergenic | 89810 | NA | 7 | 117972595 |
| 10:106457576:A:G | Indirect effect | 14.63 | SORCS3 | intronic | 0 | NA | 10 | 106457576 |
| 5:154833529:A:G | Indirect effect | 14.62 | CTB-95D12.1 | intergenic | 37367 | NA | 5 | 154833529 |
| 5:165750226:A:G | Indirect effect | 14.6 | CTB-63M22.1 | intergenic | 59084 | NA | 5 | 165750226 |
| 2:145958782:T:TA | Indirect effect | 14.58 | TEX41 | intergenic | 18566 | NA | 2 | 145958782 |
| 4:35552506:T:TA | Indirect effect | 14.58 | RNU6-573P | intergenic | 54881 | NA | 4 | 35552506 |
| 4:147865366:C:T | Indirect effect | 14.57 | RP11-292D4.2 | ncRNA_intronic | 0 | NA | 4 | 147865366 |
| 10:106556804:G:T | Indirect effect | 14.5 | SORCS3 | intronic | 0 | NA | 10 | 106556804 |
| 7:114300717:C:T | Indirect effect | 14.5 | FOXP2 | intronic | 0 | NA | 7 | 114300717 |
| 1:73851675:C:T | Indirect effect | 14.48 | RP4-598G3.1 | intergenic | 30741 | NA | 1 | 73851675 |
| 11:112983139:A:T | Indirect effect | 14.46 | NCAM1 | intronic | 0 | NA | 11 | 112983139 |
| 5:154829195:C:G | Indirect effect | 14.44 | CTB-95D12.1 | intergenic | 41701 | NA | 5 | 154829195 |
| 10:106602496:C:CT | Indirect effect | 14.43 | SORCS3 | intronic | 0 | NA | 10 | 106602496 |
| 4:147771978:A:T | Indirect effect | 14.42 | TTC29 | intronic | 0 | NA | 4 | 147771978 |
| 5:154872210:G:T | Indirect effect | 14.41 | CTB-95D12.1 | ncRNA_exonic | 0 | NA | 5 | 154872210 |
| 10:104418350:C:T | Indirect effect | 14.4 | TRIM8 | downstream | 0 | NA | 10 | 104418350 |
| 11:17161615:C:T | Indirect effect | 14.38 | PIK3C2A | intronic | 0 | NA | 11 | 17161615 |
| 15:47916872:C:G | Indirect effect | 14.38 | SEMA6D | intronic | 0 | NA | 15 | 47916872 |
| 10:104814367:C:T | Indirect effect | 14.37 | CNNM2 | intronic | 0 | NA | 10 | 104814367 |
| 2:146258263:C:T | Indirect effect | 14.37 | AC092484.1 | intergenic | 68599 | NA | 2 | 146258263 |
| 4:147824864:A:G | Indirect effect | 14.35 | TTC29 | exonic | 0 | nonsynonymous SNV | 4 | 147824864 |
| 4:147923616:C:T | Indirect effect | 14.32 | RP11-292D4.3 | intergenic | 3028 | NA | 4 | 147923616 |
| 7:32292961:A:T | Indirect effect | 14.28 | PDE1C | intronic | 0 | NA | 7 | 32292961 |
| 10:104456725:G:T | Indirect effect | 14.22 | ARL3 | intronic | 0 | NA | 10 | 104456725 |
| 3:50209053:G:T | Indirect effect | 14.19 | SEMA3F | intronic | 0 | NA | 3 | 50209053 |
| 9:127913980:G:T | Indirect effect | 14.14 | PPP6C | intronic | 0 | NA | 9 | 127913980 |
| 4:35603356:A:G | Indirect effect | 14.11 | RNU6-573P | intergenic | 105731 | NA | 4 | 35603356 |
| 8:92021036:C:T | Indirect effect | 14.11 | TMEM55A | intronic | 0 | NA | 8 | 92021036 |
| 4:147683357:A:G | Indirect effect | 14.07 | TTC29 | intronic | 0 | NA | 4 | 147683357 |
| 4:35599866:C:T | Indirect effect | 14.06 | RNU6-573P | intergenic | 102241 | NA | 4 | 35599866 |
| 3:50138580:C:CTAGT | Indirect effect | 14.03 | RBM5 | UTR3 | 0 | NA | 3 | 50138580 |
| 5:165689727:A:C | Indirect effect | 14.03 | AC122720.1 | intergenic | 103202 | NA | 5 | 165689727 |
| 9:128153244:A:G | Indirect effect | 14.02 | RP11-184B22.2 | intergenic | 18199 | NA | 9 | 128153244 |
| 11:28680143:C:T | Indirect effect | 14.01 | RP11-960D24.1 | intergenic | 20587 | NA | 11 | 28680143 |
| 2:32036625:A:G | Indirect effect | 14.01 | AL121652.2 | intergenic | 1268 | NA | 2 | 32036625 |
| 4:147802718:A:G | Indirect effect | 13.99 | TTC29 | intronic | 0 | NA | 4 | 147802718 |
| 4:35575416:C:T | Indirect effect | 13.96 | RNU6-573P | intergenic | 77791 | NA | 4 | 35575416 |
| 15:78963994:A:G | Direct effect | 13.95 | RP11-160C18.2 | ncRNA_intronic | 0 | NA | 15 | 78963994 |
| 9:128723226:A:T | Indirect effect | 13.95 | PBX3 | intronic | 0 | NA | 9 | 128723226 |
| 3:49571462:G:T | Indirect effect | 13.88 | DAG1 | UTR3 | 0 | NA | 3 | 49571462 |
| 2:146357454:T:TTG | Indirect effect | 13.87 | AC079163.1 | intergenic | 1021 | NA | 2 | 146357454 |
| 3:49275805:A:G | Indirect effect | 13.86 | CCDC36 | intronic | 0 | NA | 3 | 49275805 |
| 9:127928735:C:T | Indirect effect | 13.85 | PPP6C | intronic | 0 | NA | 9 | 127928735 |
| 11:112839532:A:G | Indirect effect | 13.84 | NCAM1 | intronic | 0 | NA | 11 | 112839532 |
| 3:50126215:A:C | Indirect effect | 13.83 | RBM6 | intronic | 0 | NA | 3 | 50126215 |
| 9:127957514:C:G | Indirect effect | 13.83 | RP11-366O20.5 | ncRNA_exonic | 0 | NA | 9 | 127957514 |
| 10:104855278:C:T | Indirect effect | 13.82 | NT5C2 | intronic | 0 | NA | 10 | 104855278 |
| 9:3014254:C:T | Indirect effect | 13.82 | CARM1P1 | ncRNA_intronic | 0 | NA | 9 | 3014254 |
| 10:8792629:A:T | Indirect effect | 13.77 | RP11-575N15.1 | intergenic | 26378 | NA | 10 | 8792629 |
| 8:42633395:A:AG | Indirect effect | 13.77 | CHRNA6 | intronic | 0 | NA | 8 | 42633395 |
| 11:17094258:C:T | Indirect effect | 13.76 | AC116533.1 | intergenic | 1241 | NA | 11 | 17094258 |
| 2:186031374:T:TA | Indirect effect | 13.75 | ZNF804A | intergenic | 227155 | NA | 2 | 186031374 |
| 7:114071035:C:T | Indirect effect | 13.72 | FOXP2 | intronic | 0 | NA | 7 | 114071035 |
| 9:128435126:A:C | Indirect effect | 13.72 | MAPKAP1 | intronic | 0 | NA | 9 | 128435126 |
| 5:165748003:C:T | Indirect effect | 13.71 | CTB-63M22.1 | intergenic | 61307 | NA | 5 | 165748003 |
| 18:35145840:T:TCCTCTCTCTCTC | Indirect effect | 13.7 | CELF4 | UTR5 | 0 | NA | 18 | 35145840 |
| 7:118072054:C:G | Indirect effect | 13.69 | AC006926.1 | intergenic | 30499 | NA | 7 | 118072054 |
| 3:49443081:C:T | Indirect effect | 13.67 | RHOA | intronic | 0 | NA | 3 | 49443081 |
| 15:47939956:A:G | Indirect effect | 13.65 | SEMA6D | intronic | 0 | NA | 15 | 47939956 |
| 3:50144951:C:T | Indirect effect | 13.65 | RBM5 | exonic | 0 | synonymous SNV | 3 | 50144951 |
| 4:35558247:A:G | Indirect effect | 13.65 | RNU6-573P | intergenic | 60622 | NA | 4 | 35558247 |
| 10:106463771:A:G | Indirect effect | 13.6 | SORCS3 | intronic | 0 | NA | 10 | 106463771 |
| 17:27588806:A:C | Indirect effect | 13.59 | NUFIP2 | UTR3 | 0 | NA | 17 | 27588806 |
| 9:128424508:A:G | Indirect effect | 13.59 | MAPKAP1 | intronic | 0 | NA | 9 | 128424508 |
| 15:79112622:G:T | Direct effect | 13.58 | MORF4L1 | intronic | 0 | NA | 15 | 79112622 |
| 18:35131444:A:G | Indirect effect | 13.56 | CELF4 | intronic | 0 | NA | 18 | 35131444 |
| 3:49040735:C:CTGTGGCTGGA | Indirect effect | 13.56 | P4HTM | intronic | 0 | NA | 3 | 49040735 |
| 2:32347785:A:G | Indirect effect | 13.55 | SPAST | intronic | 0 | NA | 2 | 32347785 |
| 10:106534343:C:G | Indirect effect | 13.53 | SORCS3 | intronic | 0 | NA | 10 | 106534343 |
| 11:112980929:A:AT | Indirect effect | 13.53 | NCAM1 | intronic | 0 | NA | 11 | 112980929 |
| 7:118048504:C:T | Indirect effect | 13.5 | AC006926.1 | intergenic | 54049 | NA | 7 | 118048504 |
| 3:50040194:A:T | Indirect effect | 13.48 | RBM6 | intronic | 0 | NA | 3 | 50040194 |
| 5:166994057:T:TA | Indirect effect | 13.48 | TENM2 | intronic | 0 | NA | 5 | 166994057 |
| 15:47494272:C:T | Indirect effect | 13.45 | SEMA6D | intronic | 0 | NA | 15 | 47494272 |
| 15:47848309:C:G | Indirect effect | 13.42 | SEMA6D | intronic | 0 | NA | 15 | 47848309 |
| 4:35549859:C:T | Indirect effect | 13.41 | RNU6-573P | intergenic | 52234 | NA | 4 | 35549859 |
| 9:128723227:C:G | Indirect effect | 13.38 | PBX3 | intronic | 0 | NA | 9 | 128723227 |
| 1:73851687:G:T | Indirect effect | 13.37 | RP4-598G3.1 | intergenic | 30753 | NA | 1 | 73851687 |
| 15:79023578:A:G | Direct effect | 13.36 | RP11-160C18.2 | ncRNA_intronic | 0 | NA | 15 | 79023578 |
| 1:50591851:A:G | Indirect effect | 13.3 | ELAVL4 | intronic | 0 | NA | 1 | 50591851 |
| 8:27435201:C:T | Indirect effect | 13.3 | GULOP | ncRNA_exonic | 0 | NA | 8 | 27435201 |
| 8:27426077:A:T | Indirect effect | 13.28 | GULOP | ncRNA_intronic | 0 | NA | 8 | 27426077 |
| 5:167022878:C:T | Indirect effect | 13.26 | TENM2 | intronic | 0 | NA | 5 | 167022878 |
| 2:105977776:A:G | Indirect effect | 13.23 | FHL2 | exonic | 0 | synonymous SNV | 2 | 105977776 |
| 2:22604358:C:G | Indirect effect | 13.23 | AC068490.2 | ncRNA_intronic | 0 | NA | 2 | 22604358 |
| 7:114145525:A:G | Indirect effect | 13.23 | FOXP2 | intronic | 0 | NA | 7 | 114145525 |
| 7:117784427:A:T | Indirect effect | 13.23 | NAA38 | intergenic | 39659 | NA | 7 | 117784427 |
| 4:35519558:A:G | Indirect effect | 13.22 | RNU6-573P | intergenic | 21933 | NA | 4 | 35519558 |
| 18:53253423:C:G | Indirect effect | 13.21 | TCF4 | intronic | 0 | NA | 18 | 53253423 |
| 18:35200180:A:G | Indirect effect | 13.2 | MIR4318 | intergenic | 36918 | NA | 18 | 35200180 |
| 10:104916857:C:T | Indirect effect | 13.19 | NT5C2 | intronic | 0 | NA | 10 | 104916857 |
| 2:32016677:A:G | Indirect effect | 13.19 | AL133249.1 | intergenic | 2215 | NA | 2 | 32016677 |
| 2:32440868:G:T | Indirect effect | 13.18 | SLC30A6 | intronic | 0 | NA | 2 | 32440868 |
| 3:49395673:G:GGCC | Indirect effect | 13.18 | GPX1 | exonic | 0 | NA | 3 | 49395673 |
| 2:203729763:A:G | Indirect effect | 13.17 | ICA1L | intronic | 0 | NA | 2 | 203729763 |
| 3:49961780:A:G | Indirect effect | 13.15 | MON1A | intronic | 0 | NA | 3 | 49961780 |
| 10:104241983:C:T | Indirect effect | 13.1 | ACTR1A | intronic | 0 | NA | 10 | 104241983 |
| 4:35549864:C:T | Indirect effect | 13.1 | RNU6-573P | intergenic | 52239 | NA | 4 | 35549864 |
| 10:8785995:C:T | Indirect effect | 13.09 | RP11-575N15.1 | intergenic | 19744 | NA | 10 | 8785995 |
| 15:79031290:A:G | Direct effect | 13.09 | RP11-160C18.2 | intergenic | 3453 | NA | 15 | 79031290 |
| 9:128199873:C:T | Indirect effect | 13.09 | MAPKAP1 | UTR3 | 0 | NA | 9 | 128199873 |
| 1:44052198:A:G | Indirect effect | 13.08 | PTPRF | intronic | 0 | NA | 1 | 44052198 |
| 15:79031138:A:C | Direct effect | 13.07 | RP11-160C18.2 | intergenic | 3301 | NA | 15 | 79031138 |
| 8:42817771:A:AC | Indirect effect | 13.07 | HOOK3 | intronic | 0 | NA | 8 | 42817771 |
| 7:117925336:A:T | Indirect effect | 13.06 | ANKRD7 | intergenic | 42551 | NA | 7 | 117925336 |
| 3:49584745:A:G | Indirect effect | 13.05 | BSN-AS2 | intergenic | 1994 | NA | 3 | 49584745 |
| 15:47644157:C:G | Indirect effect | 13.03 | SEMA6D | intronic | 0 | NA | 15 | 47644157 |
| 9:127910307:G:T | Indirect effect | 13.03 | PPP6C | UTR3 | 0 | NA | 9 | 127910307 |
| 9:128141348:A:G | Indirect effect | 13.03 | RP11-184B22.2 | intergenic | 6303 | NA | 9 | 128141348 |
| 18:50709981:A:G | Indirect effect | 13.01 | DCC | intronic | 0 | NA | 18 | 50709981 |
| 2:146352036:A:C | Indirect effect | 12.99 | AC079163.1 | intergenic | 6439 | NA | 2 | 146352036 |
| 1:44076019:A:G | Indirect effect | 12.97 | PTPRF | intronic | 0 | NA | 1 | 44076019 |
| 1:73860311:A:C | Indirect effect | 12.97 | RP4-598G3.1 | intergenic | 39377 | NA | 1 | 73860311 |
| 2:203969504:A:G | Indirect effect | 12.95 | NBEAL1 | intronic | 0 | NA | 2 | 203969504 |
| 9:128277258:A:G | Indirect effect | 12.95 | MAPKAP1 | intronic | 0 | NA | 9 | 128277258 |
| 15:79031139:C:G | Direct effect | 12.94 | RP11-160C18.2 | intergenic | 3302 | NA | 15 | 79031139 |
| 4:123266637:G:GTGAACATTTAATTGGTTGGAGCCAATTAAATGT | Indirect effect | 12.94 | KIAA1109 | intronic | 0 | NA | 4 | 123266637 |
| 9:127913977:C:CT | Indirect effect | 12.94 | PPP6C | intronic | 0 | NA | 9 | 127913977 |
| 15:47498128:T:TTTATC | Indirect effect | 12.9 | SEMA6D | intronic | 0 | NA | 15 | 47498128 |
| 15:47679524:C:T | Indirect effect | 12.9 | CTD-2050N2.1 | ncRNA_intronic | 0 | NA | 15 | 47679524 |
| 5:167006009:C:T | Indirect effect | 12.89 | TENM2 | intronic | 0 | NA | 5 | 167006009 |
| 5:165727304:C:G | Indirect effect | 12.87 | CTB-63M22.1 | intergenic | 82006 | NA | 5 | 165727304 |
| 3:49143438:A:C | Indirect effect | 12.85 | QARS | upstream | 0 | NA | 3 | 49143438 |
| 10:104454113:C:G | Indirect effect | 12.82 | ARL3 | intronic | 0 | NA | 10 | 104454113 |
| 11:28628549:A:C | Indirect effect | 12.81 | RP11-960D24.1 | intergenic | 72181 | NA | 11 | 28628549 |
| 15:78796732:C:T | Direct effect | 12.8 | IREB2 | intergenic | 2934 | NA | 15 | 78796732 |
| 15:79031079:C:T | Direct effect | 12.8 | RP11-160C18.2 | intergenic | 3242 | NA | 15 | 79031079 |
| 2:127716770:A:G | Indirect effect | 12.78 | AC114783.1 | intergenic | 57097 | NA | 2 | 127716770 |
| 7:117510621:C:T | Indirect effect | 12.78 | CTTNBP2 | intronic | 0 | NA | 7 | 117510621 |
| 10:106611268:A:G | Indirect effect | 12.76 | SORCS3 | intronic | 0 | NA | 10 | 106611268 |
| 2:204056678:C:T | Indirect effect | 12.76 | NBEAL1 | intronic | 0 | NA | 2 | 204056678 |
| 8:91886258:A:T | Indirect effect | 12.73 | NECAB1 | intronic | 0 | NA | 8 | 91886258 |
| 9:128563948:C:CT | Indirect effect | 12.72 | PBX3 | intronic | 0 | NA | 9 | 128563948 |
| 15:47683596:G:GC | Indirect effect | 12.69 | CTD-2050N2.1 | ncRNA_intronic | 0 | NA | 15 | 47683596 |
| 19:41352936:C:T | Indirect effect | 12.68 | CYP2A6 | exonic | 0 | synonymous SNV | 19 | 41352936 |
| 5:166987674:G:T | Indirect effect | 12.66 | TENM2 | intronic | 0 | NA | 5 | 166987674 |
| 15:79117917:A:T | Direct effect | 12.63 | MORF4L1 | intronic | 0 | NA | 15 | 79117917 |
| 9:128441641:C:T | Indirect effect | 12.63 | MAPKAP1 | intronic | 0 | NA | 9 | 128441641 |
| 14:80358262:G:T | Indirect effect | 12.62 | RP11-526N18.1 | intergenic | 1161 | NA | 14 | 80358262 |
| 9:128522366:C:G | Indirect effect | 12.6 | PBX3 | intronic | 0 | NA | 9 | 128522366 |
| 9:136474498:G:T | Indirect effect | 12.6 | LL09NC01-254D11.1 | intergenic | 2422 | NA | 9 | 136474498 |
| 15:47547177:C:T | Indirect effect | 12.59 | SEMA6D | intronic | 0 | NA | 15 | 47547177 |
| 15:79023577:C:T | Direct effect | 12.59 | RP11-160C18.2 | ncRNA_intronic | 0 | NA | 15 | 79023577 |
| 2:31805706:C:G | Indirect effect | 12.57 | SRD5A2 | ncRNA_exonic | 0 | NA | 2 | 31805706 |
| 15:79030881:A:G | Direct effect | 12.56 | RP11-160C18.2 | intergenic | 3044 | NA | 15 | 79030881 |
| 19:41356379:A:C | Indirect effect | 12.56 | CTC-490E21.12 | intronic | 0 | NA | 19 | 41356379 |
| 7:115076026:C:T | Indirect effect | 12.55 | RP11-222O23.1 | intergenic | 67395 | NA | 7 | 115076026 |
| 7:117825681:A:G | Indirect effect | 12.55 | NAA38 | UTR5 | 0 | NA | 7 | 117825681 |
| 2:22587157:A:C | Indirect effect | 12.52 | AC068490.2 | ncRNA_intronic | 0 | NA | 2 | 22587157 |
| 3:49949071:A:G | Indirect effect | 12.52 | MON1A | exonic | 0 | synonymous SNV | 3 | 49949071 |
| 4:35520456:A:G | Indirect effect | 12.51 | RNU6-573P | intergenic | 22831 | NA | 4 | 35520456 |
| 3:49899795:A:G | Indirect effect | 12.49 | CAMKV | exonic | 0 | synonymous SNV | 3 | 49899795 |
| 2:32343167:A:T | Indirect effect | 12.47 | SPAST | intronic | 0 | NA | 2 | 32343167 |
| 7:114060663:A:T | Indirect effect | 12.47 | FOXP2 | intronic | 0 | NA | 7 | 114060663 |
| 11:17321422:G:T | Indirect effect | 12.46 | NUCB2 | intronic | 0 | NA | 11 | 17321422 |
| 9:128655872:A:ATTGT | Indirect effect | 12.45 | PBX3 | intronic | 0 | NA | 9 | 128655872 |
| 9:128728395:T:TA | Indirect effect | 12.45 | PBX3 | UTR3 | 0 | NA | 9 | 128728395 |
| 10:8811233:A:G | Indirect effect | 12.43 | RP11-575N15.1 | intergenic | 44982 | NA | 10 | 8811233 |
| 4:35552163:A:T | Indirect effect | 12.43 | RNU6-573P | intergenic | 54538 | NA | 4 | 35552163 |
| 5:154958513:C:T | Indirect effect | 12.43 | CTB-95D12.1 | intergenic | 85355 | NA | 5 | 154958513 |
| 2:32447868:A:G | Indirect effect | 12.42 | SLC30A6 | UTR3 | 0 | NA | 2 | 32447868 |
| 7:117615317:C:T | Indirect effect | 12.38 | AC003084.2 | intergenic | 23595 | NA | 7 | 117615317 |
| 7:1876081:C:T | Indirect effect | 12.38 | MAD1L1 | intronic | 0 | NA | 7 | 1876081 |
| 14:69752420:C:T | Indirect effect | 12.37 | GALNT16 | intronic | 0 | NA | 14 | 69752420 |
| 1:44207553:C:T | Indirect effect | 12.36 | ST3GAL3 | intronic | 0 | NA | 1 | 44207553 |
| 2:203828192:A:G | Indirect effect | 12.36 | CARF | intronic | 0 | NA | 2 | 203828192 |
| 5:154855002:G:T | Indirect effect | 12.36 | CTB-95D12.1 | intergenic | 15894 | NA | 5 | 154855002 |
| 18:50723460:C:T | Indirect effect | 12.33 | DCC | intronic | 0 | NA | 18 | 50723460 |
| 15:78007213:C:T | Indirect effect | 12.31 | LINGO1 | intronic | 0 | NA | 15 | 78007213 |
| 10:106535179:A:G | Indirect effect | 12.3 | SORCS3 | intronic | 0 | NA | 10 | 106535179 |
| 7:117623182:A:G | Indirect effect | 12.3 | AC003084.2 | intergenic | 15730 | NA | 7 | 117623182 |
| 15:79112568:A:C | Direct effect | 12.29 | MORF4L1 | intronic | 0 | NA | 15 | 79112568 |
| 15:79031485:A:G | Direct effect | 12.27 | RP11-160C18.2 | intergenic | 3648 | NA | 15 | 79031485 |
| 15:47554562:A:G | Indirect effect | 12.24 | SEMA6D | intronic | 0 | NA | 15 | 47554562 |
| 4:147923166:C:T | Indirect effect | 12.24 | RP11-292D4.3 | intergenic | 3478 | NA | 4 | 147923166 |
| 7:114302833:G:T | Indirect effect | 12.24 | FOXP2 | intronic | 0 | NA | 7 | 114302833 |
| 7:118182503:C:T | Indirect effect | 12.24 | AC006926.1 | intergenic | 79855 | NA | 7 | 118182503 |
| 9:128649078:T:TACAACTTGA | Indirect effect | 12.23 | PBX3 | intronic | 0 | NA | 9 | 128649078 |
| 2:32289746:C:T | Indirect effect | 12.22 | SPAST | intronic | 0 | NA | 2 | 32289746 |
| 9:127913981:C:G | Indirect effect | 12.22 | PPP6C | intronic | 0 | NA | 9 | 127913981 |
| 7:118017925:C:T | Indirect effect | 12.2 | AC006926.1 | intergenic | 84628 | NA | 7 | 118017925 |
| 10:106534317:A:G | Indirect effect | 12.19 | SORCS3 | intronic | 0 | NA | 10 | 106534317 |
| 15:78944718:A:G | Direct effect | 12.19 | CHRNB4 | intronic | 0 | NA | 15 | 78944718 |
| 2:146217925:A:G | Indirect effect | 12.17 | AC092484.1 | intergenic | 108937 | NA | 2 | 146217925 |
| 2:203792628:A:G | Indirect effect | 12.17 | CARF | intronic | 0 | NA | 2 | 203792628 |
| 14:69652125:A:T | Indirect effect | 12.16 | RP11-363J20.1 | ncRNA_intronic | 0 | NA | 14 | 69652125 |
| 15:47556977:G:T | Indirect effect | 12.16 | SEMA6D | intronic | 0 | NA | 15 | 47556977 |
| 7:118196744:C:T | Indirect effect | 12.16 | AC006926.1 | intergenic | 94096 | NA | 7 | 118196744 |
| 2:61824932:C:G | Indirect effect | 12.14 | RNU6-1145P | intergenic | 7819 | NA | 2 | 61824932 |
| 3:48784270:A:G | Indirect effect | 12.14 | PRKAR2A | UTR3 | 0 | NA | 3 | 48784270 |
| 15:47796089:C:T | Indirect effect | 12.12 | SEMA6D | intronic | 0 | NA | 15 | 47796089 |
| 9:128314394:C:T | Indirect effect | 12.12 | MAPKAP1 | intronic | 0 | NA | 9 | 128314394 |
| 11:112834153:C:T | Indirect effect | 12.11 | RP11-629G13.1 | ncRNA_exonic | 0 | NA | 11 | 112834153 |
| 4:123329362:A:G | Indirect effect | 12.11 | ADAD1 | intronic | 0 | NA | 4 | 123329362 |
| 2:145944007:C:T | Indirect effect | 12.1 | TEX41 | intergenic | 3791 | NA | 2 | 145944007 |
| 15:47569588:G:T | Indirect effect | 12.09 | SEMA6D | intronic | 0 | NA | 15 | 47569588 |
| 14:30066929:A:G | Indirect effect | 12.08 | PRKD1 | exonic | 0 | synonymous SNV | 14 | 30066929 |
| 7:1874478:A:G | Indirect effect | 12.08 | MAD1L1 | intronic | 0 | NA | 7 | 1874478 |
| 4:140928557:A:C | Indirect effect | 12.07 | MAML3 | intronic | 0 | NA | 4 | 140928557 |
| 17:27424316:A:G | Indirect effect | 12.06 | MYO18A | exonic | 0 | synonymous SNV | 17 | 27424316 |
| 15:79123631:C:T | Direct effect | 12.05 | MORF4L1 | intronic | 0 | NA | 15 | 79123631 |
| 2:31806031:C:G | Indirect effect | 12.04 | SRD5A2 | ncRNA_exonic | 0 | NA | 2 | 31806031 |
| 15:47844059:C:T | Indirect effect | 12.03 | SEMA6D | intronic | 0 | NA | 15 | 47844059 |
| 5:154859592:G:T | Indirect effect | 12.03 | CTB-95D12.1 | intergenic | 11304 | NA | 5 | 154859592 |
| 7:114337615:A:G | Indirect effect | 12.03 | FOXP2 | intergenic | 3788 | NA | 7 | 114337615 |
| 7:114332246:G:T | Indirect effect | 11.99 | FOXP2 | UTR3 | 0 | NA | 7 | 114332246 |
| 2:61454059:A:T | Indirect effect | 11.98 | USP34 | UTR3 | 0 | NA | 2 | 61454059 |
| 8:91907491:C:T | Indirect effect | 11.96 | NECAB1 | intronic | 0 | NA | 8 | 91907491 |
| 1:73870901:A:G | Indirect effect | 11.95 | RP4-598G3.1 | intergenic | 49967 | NA | 1 | 73870901 |
| 9:128295198:A:G | Indirect effect | 11.95 | MAPKAP1 | intronic | 0 | NA | 9 | 128295198 |
| 7:1852166:A:G | Indirect effect | 11.94 | MAD1L1 | intergenic | 3264 | NA | 7 | 1852166 |
| 9:128719849:A:G | Indirect effect | 11.94 | PBX3 | intronic | 0 | NA | 9 | 128719849 |
| 7:118082475:T:TA | Indirect effect | 11.93 | NA | NA | NA | NA | NA | NA |
| 4:147732785:C:T | Indirect effect | 11.92 | TTC29 | intronic | 0 | NA | 4 | 147732785 |
| 1:73926572:C:T | Indirect effect | 11.91 | RP4-598G3.1 | intergenic | 105638 | NA | 1 | 73926572 |
| 7:118082475:T:TAA | Indirect effect | 11.9 | AC006926.1 | intergenic | 20078 | NA | 7 | 118082475 |
| 15:79123505:A:G | Direct effect | 11.89 | MORF4L1 | intronic | 0 | NA | 15 | 79123505 |
| 1:50585283:A:C | Indirect effect | 11.89 | ELAVL4 | intronic | 0 | NA | 1 | 50585283 |
| 2:32476390:G:T | Indirect effect | 11.89 | NLRC4 | exonic | 0 | synonymous SNV | 2 | 32476390 |
| 2:45128493:A:G | Indirect effect | 11.89 | RP11-89K21.1 | intergenic | 18839 | NA | 2 | 45128493 |
| 9:128233058:G:T | Indirect effect | 11.89 | MAPKAP1 | intronic | 0 | NA | 9 | 128233058 |
| 3:49298205:C:T | Indirect effect | 11.88 | RP11-3B7.1 | exonic | 0 | nonsynonymous SNV | 3 | 49298205 |
| 4:147847149:C:G | Indirect effect | 11.88 | TTC29 | intronic | 0 | NA | 4 | 147847149 |
| 5:154878884:A:G | Indirect effect | 11.88 | CTB-95D12.1 | intergenic | 5726 | NA | 5 | 154878884 |
| 15:79022432:G:GTAGTGCTCCCC | Direct effect | 11.87 | RP11-160C18.2 | ncRNA_intronic | 0 | NA | 15 | 79022432 |
| 15:79112574:A:G | Direct effect | 11.87 | MORF4L1 | intronic | 0 | NA | 15 | 79112574 |
| 11:17050008:A:G | Indirect effect | 11.85 | OR7E14P | ncRNA_intronic | 0 | NA | 11 | 17050008 |
| 15:79022420:C:T | Direct effect | 11.85 | RP11-160C18.2 | ncRNA_intronic | 0 | NA | 15 | 79022420 |
| 5:154778196:A:G | Indirect effect | 11.85 | CTC-447K7.1 | downstream | 0 | NA | 5 | 154778196 |
| 5:165749891:A:G | Indirect effect | 11.83 | CTB-63M22.1 | intergenic | 59419 | NA | 5 | 165749891 |
| 15:79123753:C:T | Direct effect | 11.82 | MORF4L1 | intronic | 0 | NA | 15 | 79123753 |
| 4:35581837:G:T | Indirect effect | 11.82 | RNU6-573P | intergenic | 84212 | NA | 4 | 35581837 |
| 9:128319066:A:G | Indirect effect | 11.81 | MAPKAP1 | intronic | 0 | NA | 9 | 128319066 |
| 7:117937140:A:G | Indirect effect | 11.79 | ANKRD7 | intergenic | 54355 | NA | 7 | 117937140 |
| 2:203670122:C:T | Indirect effect | 11.78 | ICA1L | intronic | 0 | NA | 2 | 203670122 |
| 10:106632877:C:T | Indirect effect | 11.76 | SORCS3 | intronic | 0 | NA | 10 | 106632877 |
| 18:50723283:G:T | Indirect effect | 11.76 | DCC | intronic | 0 | NA | 18 | 50723283 |
| 1:50379591:A:C | Indirect effect | 11.72 | AGBL4 | intronic | 0 | NA | 1 | 50379591 |
| 10:105142179:A:G | Indirect effect | 11.68 | TAF5 | intronic | 0 | NA | 10 | 105142179 |
| 10:104350225:A:G | Indirect effect | 11.67 | SUFU | intronic | 0 | NA | 10 | 104350225 |
| 15:78807872:C:T | Direct effect | 11.67 | HYKK | intronic | 0 | NA | 15 | 78807872 |
| 3:49948728:C:T | Indirect effect | 11.66 | MON1A | intronic | 0 | NA | 3 | 49948728 |
| 5:154865176:C:CTG | Indirect effect | 11.66 | CTB-95D12.1 | intergenic | 5720 | NA | 5 | 154865176 |
| 9:128222314:T:TC | Indirect effect | 11.66 | MAPKAP1 | intronic | 0 | NA | 9 | 128222314 |
| 11:17293462:A:T | Indirect effect | 11.65 | NUCB2 | intronic | 0 | NA | 11 | 17293462 |
| 3:50022926:C:T | Indirect effect | 11.64 | RBM6 | intronic | 0 | NA | 3 | 50022926 |
| 15:47597049:A:C | Indirect effect | 11.63 | SEMA6D | intronic | 0 | NA | 15 | 47597049 |
| 3:49213637:C:T | Indirect effect | 11.63 | KLHDC8B | UTR3 | 0 | NA | 3 | 49213637 |
| 2:161283525:A:G | Indirect effect | 11.61 | RBMS1 | intronic | 0 | NA | 2 | 161283525 |
| 7:117668235:A:T | Indirect effect | 11.61 | AC003084.2 | intergenic | 24136 | NA | 7 | 117668235 |
| 7:118139824:C:T | Indirect effect | 11.61 | AC006926.1 | intergenic | 37176 | NA | 7 | 118139824 |
| 9:128278687:C:T | Indirect effect | 11.61 | MAPKAP1 | intronic | 0 | NA | 9 | 128278687 |
| 15:79051721:A:G | Direct effect | 11.6 | ADAMTS7 | UTR3 | 0 | NA | 15 | 79051721 |
| 7:117856291:A:G | Indirect effect | 11.6 | ANKRD7 | intergenic | 8421 | NA | 7 | 117856291 |
| 15:79123779:C:T | Direct effect | 11.57 | MORF4L1 | intronic | 0 | NA | 15 | 79123779 |
| 1:73851936:A:G | Indirect effect | 11.57 | RP4-598G3.1 | intergenic | 31002 | NA | 1 | 73851936 |
| 2:32341408:A:G | Indirect effect | 11.57 | SPAST | intronic | 0 | NA | 2 | 32341408 |
| 3:49902544:A:G | Indirect effect | 11.57 | CAMKV | intronic | 0 | NA | 3 | 49902544 |
| 9:127893864:C:T | Indirect effect | 11.56 | SCAI | intronic | 0 | NA | 9 | 127893864 |
| 15:47839785:G:T | Indirect effect | 11.55 | SEMA6D | intronic | 0 | NA | 15 | 47839785 |
| 5:154844317:T:TA | Indirect effect | 11.55 | CTB-95D12.1 | intergenic | 26578 | NA | 5 | 154844317 |
| 1:44173423:A:G | Indirect effect | 11.54 | KDM4A-AS1 | ncRNA_intronic | 0 | NA | 1 | 44173423 |
| 15:78898932:C:G | Direct effect | 11.53 | CHRNA3 | intronic | 0 | NA | 15 | 78898932 |
| 5:154857774:C:T | Indirect effect | 11.53 | CTB-95D12.1 | intergenic | 13122 | NA | 5 | 154857774 |
| 1:96163412:C:T | Indirect effect | 11.52 | RP11-286B14.1 | ncRNA_intronic | 0 | NA | 1 | 96163412 |
| 9:128489937:A:G | Indirect effect | 11.52 | RP11-423C15.3 | intergenic | 16096 | NA | 9 | 128489937 |
| 10:104434630:A:C | Indirect effect | 11.51 | ARL3 | UTR3 | 0 | NA | 10 | 104434630 |
| 15:79045517:A:G | Direct effect | 11.5 | RP11-160C18.4 | ncRNA_exonic | 0 | NA | 15 | 79045517 |
| 15:78799060:A:ACT | Direct effect | 11.49 | HYKK | upstream | 0 | NA | 15 | 78799060 |
| 11:17348878:G:T | Indirect effect | 11.48 | NUCB2 | intronic | 0 | NA | 11 | 17348878 |
| 15:47636386:A:AT | Indirect effect | 11.48 | SEMA6D | intronic | 0 | NA | 15 | 47636386 |
| 3:50128386:A:G | Indirect effect | 11.48 | RBM5 | intronic | 0 | NA | 3 | 50128386 |
| 15:79023433:A:G | Direct effect | 11.47 | RP11-160C18.2 | ncRNA_intronic | 0 | NA | 15 | 79023433 |
| 19:21219018:A:G | Indirect effect | 11.47 | ZNF430 | UTR3 | 0 | NA | 19 | 21219018 |
| 9:128112896:A:G | Indirect effect | 11.47 | GAPVD1 | intronic | 0 | NA | 9 | 128112896 |
| 9:128729703:A:G | Indirect effect | 11.47 | PBX3 | downstream | 0 | NA | 9 | 128729703 |
| 10:122195629:A:G | Indirect effect | 11.46 | RP11-572P18.2 | ncRNA_exonic | 0 | NA | 10 | 122195629 |
| 9:128668715:A:G | Indirect effect | 11.46 | PBX3 | intronic | 0 | NA | 9 | 128668715 |
| 11:28667940:C:T | Indirect effect | 11.45 | RP11-960D24.1 | intergenic | 32790 | NA | 11 | 28667940 |
| 15:47933007:A:G | Indirect effect | 11.44 | SEMA6D | intronic | 0 | NA | 15 | 47933007 |
| 15:79029396:C:T | Direct effect | 11.44 | RP11-160C18.2 | intergenic | 1559 | NA | 15 | 79029396 |
| 11:17296463:A:C | Indirect effect | 11.41 | NUCB2 | intronic | 0 | NA | 11 | 17296463 |
| 2:161346379:C:T | Indirect effect | 11.39 | RBMS1 | intronic | 0 | NA | 2 | 161346379 |
| 7:114620506:C:T | Indirect effect | 11.39 | MDFIC | intronic | 0 | NA | 7 | 114620506 |
| 7:118182781:G:T | Indirect effect | 11.39 | AC006926.1 | intergenic | 80133 | NA | 7 | 118182781 |
| 9:128255582:A:T | Indirect effect | 11.38 | MAPKAP1 | intronic | 0 | NA | 9 | 128255582 |
| 10:8790399:C:T | Indirect effect | 11.37 | RP11-575N15.1 | intergenic | 24148 | NA | 10 | 8790399 |
| 3:49941436:A:G | Indirect effect | 11.37 | CTD-2330K9.3 | UTR5 | 0 | NA | 3 | 49941436 |
| 2:32016460:C:T | Indirect effect | 11.36 | AL133249.1 | intergenic | 2432 | NA | 2 | 32016460 |
| 7:114328160:G:T | Indirect effect | 11.36 | FOXP2 | intronic | 0 | NA | 7 | 114328160 |
| 2:186049723:A:G | Indirect effect | 11.35 | ZNF804A | intergenic | 245504 | NA | 2 | 186049723 |
| 4:140883254:C:G | Indirect effect | 11.34 | MAML3 | intronic | 0 | NA | 4 | 140883254 |
| 10:8850510:C:G | Indirect effect | 11.33 | RP11-575N15.1 | intergenic | 84259 | NA | 10 | 8850510 |
| 4:35580100:A:G | Indirect effect | 11.32 | RNU6-573P | intergenic | 82475 | NA | 4 | 35580100 |
| 10:104440050:C:T | Indirect effect | 11.3 | ARL3 | intronic | 0 | NA | 10 | 104440050 |
| 2:22442099:A:T | Indirect effect | 11.3 | AC068490.2 | ncRNA_intronic | 0 | NA | 2 | 22442099 |
| 9:128375047:A:G | Indirect effect | 11.3 | MAPKAP1 | intronic | 0 | NA | 9 | 128375047 |
| 9:128035341:C:T | Indirect effect | 11.29 | GAPVD1 | intronic | 0 | NA | 9 | 128035341 |
| 7:117543063:A:G | Indirect effect | 11.27 | AC007568.1 | intergenic | 19334 | NA | 7 | 117543063 |
| 7:117977935:A:C | Indirect effect | 11.27 | ANKRD7 | intergenic | 95150 | NA | 7 | 117977935 |
| 10:106632037:C:T | Indirect effect | 11.25 | SORCS3 | intronic | 0 | NA | 10 | 106632037 |
| 18:35152563:A:G | Indirect effect | 11.24 | CELF4 | intergenic | 6563 | NA | 18 | 35152563 |
| 7:117862242:A:G | Indirect effect | 11.24 | ANKRD7 | intergenic | 2470 | NA | 7 | 117862242 |
| 2:203714973:C:G | Indirect effect | 11.23 | ICA1L | intronic | 0 | NA | 2 | 203714973 |
| 15:79003292:C:G | Direct effect | 11.2 | RP11-160C18.2 | ncRNA_intronic | 0 | NA | 15 | 79003292 |
| 15:79026674:A:G | Direct effect | 11.2 | RP11-160C18.2 | ncRNA_intronic | 0 | NA | 15 | 79026674 |
| 8:91909980:C:T | Indirect effect | 11.2 | NECAB1 | intronic | 0 | NA | 8 | 91909980 |
| 9:128301931:C:T | Indirect effect | 11.19 | MAPKAP1 | intronic | 0 | NA | 9 | 128301931 |
| 2:203845828:A:G | Indirect effect | 11.18 | CARF | intronic | 0 | NA | 2 | 203845828 |
| 15:79026822:A:T | Direct effect | 11.17 | RP11-160C18.2 | ncRNA_intronic | 0 | NA | 15 | 79026822 |
| 5:166989242:A:G | Indirect effect | 11.15 | TENM2 | intronic | 0 | NA | 5 | 166989242 |
| 8:43092470:C:T | Indirect effect | 11.15 | RP11-726G23.2 | intergenic | 9428 | NA | 8 | 43092470 |
| 9:128067650:G:GT | Indirect effect | 11.15 | GAPVD1 | intronic | 0 | NA | 9 | 128067650 |
| 10:104734623:A:G | Indirect effect | 11.14 | CNNM2 | intronic | 0 | NA | 10 | 104734623 |
| 19:41345989:A:G | Indirect effect | 11.13 | CTC-490E21.12 | intronic | 0 | NA | 19 | 41345989 |
| 7:117965959:C:T | Indirect effect | 11.13 | ANKRD7 | intergenic | 83174 | NA | 7 | 117965959 |
| 15:79030877:C:G | Direct effect | 11.12 | RP11-160C18.2 | intergenic | 3040 | NA | 15 | 79030877 |
| 1:50589157:T:TA | Indirect effect | 11.12 | ELAVL4 | intronic | 0 | NA | 1 | 50589157 |
| 15:78929372:A:G | Direct effect | 11.11 | CHRNB4 | intronic | 0 | NA | 15 | 78929372 |
| 15:79030876:C:G | Direct effect | 11.11 | RP11-160C18.2 | intergenic | 3039 | NA | 15 | 79030876 |
| 2:145884886:C:G | Indirect effect | 11.11 | TEX41 | ncRNA_intronic | 0 | NA | 2 | 145884886 |
| 4:35501032:A:G | Indirect effect | 11.11 | RNU6-573P | intergenic | 3407 | NA | 4 | 35501032 |
| 7:1876199:C:T | Indirect effect | 11.1 | MAD1L1 | intronic | 0 | NA | 7 | 1876199 |
| 11:17324948:A:G | Indirect effect | 11.09 | NUCB2 | intronic | 0 | NA | 11 | 17324948 |
| 2:32413067:C:T | Indirect effect | 11.09 | SLC30A6 | intronic | 0 | NA | 2 | 32413067 |
| 2:185983653:C:T | Indirect effect | 11.05 | ZNF804A | intergenic | 179434 | NA | 2 | 185983653 |
| 2:203686234:G:GTTTCC | Indirect effect | 11.05 | ICA1L | intronic | 0 | NA | 2 | 203686234 |
| 11:28676139:A:G | Indirect effect | 11.04 | RP11-960D24.1 | intergenic | 24591 | NA | 11 | 28676139 |
| 15:47553048:A:T | Indirect effect | 11.03 | SEMA6D | intronic | 0 | NA | 15 | 47553048 |
| 7:117797816:A:G | Indirect effect | 11.03 | NAA38 | intergenic | 26270 | NA | 7 | 117797816 |
| 2:22443840:A:G | Indirect effect | 11.02 | AC068490.2 | ncRNA_intronic | 0 | NA | 2 | 22443840 |
| 11:17298365:C:G | Indirect effect | 11.01 | NUCB2 | UTR5 | 0 | NA | 11 | 17298365 |
| 14:69762934:C:T | Indirect effect | 11 | GALNT16 | intronic | 0 | NA | 14 | 69762934 |
| 2:203814209:C:CT | Indirect effect | 11 | CARF | intronic | 0 | NA | 2 | 203814209 |
| 9:128237178:G:T | Indirect effect | 10.97 | MAPKAP1 | intronic | 0 | NA | 9 | 128237178 |
| 3:49070499:C:T | Indirect effect | 10.95 | QRICH1 | intronic | 0 | NA | 3 | 49070499 |
| 4:35520455:C:T | Indirect effect | 10.95 | RNU6-573P | intergenic | 22830 | NA | 4 | 35520455 |
| 14:98638244:C:T | Indirect effect | 10.94 | RP11-61O1.1 | ncRNA_intronic | 0 | NA | 14 | 98638244 |
| 7:114210814:C:T | Indirect effect | 10.9 | FOXP2 | intronic | 0 | NA | 7 | 114210814 |
| 14:30066976:A:T | Indirect effect | 10.89 | PRKD1 | intronic | 0 | NA | 14 | 30066976 |
| 10:106460609:A:T | Indirect effect | 10.87 | SORCS3 | intronic | 0 | NA | 10 | 106460609 |
| 15:47637888:A:G | Indirect effect | 10.86 | SEMA6D | intronic | 0 | NA | 15 | 47637888 |
| 2:203855342:C:T | Indirect effect | 10.86 | CARF | intergenic | 3556 | NA | 2 | 203855342 |
| 7:115082406:A:G | Indirect effect | 10.86 | RP11-222O23.1 | intergenic | 61015 | NA | 7 | 115082406 |
| 2:105923080:C:T | Indirect effect | 10.85 | TGFBRAP1 | intronic | 0 | NA | 2 | 105923080 |
| 5:167032093:C:T | Indirect effect | 10.85 | TENM2 | intronic | 0 | NA | 5 | 167032093 |
| 7:118018673:A:T | Indirect effect | 10.85 | AC006926.1 | intergenic | 83880 | NA | 7 | 118018673 |
| 1:73852067:A:T | Indirect effect | 10.84 | RP4-598G3.1 | intergenic | 31133 | NA | 1 | 73852067 |
| 2:203835177:A:C | Indirect effect | 10.84 | CARF | intronic | 0 | NA | 2 | 203835177 |
| 18:77577501:T:TCA | Indirect effect | 10.82 | RP11-154H12.3 | intergenic | 1136 | NA | 18 | 77577501 |
| 7:117862220:A:G | Indirect effect | 10.81 | ANKRD7 | intergenic | 2492 | NA | 7 | 117862220 |
| 3:50035323:C:T | Indirect effect | 10.8 | RBM6 | intronic | 0 | NA | 3 | 50035323 |
| 7:114350502:A:T | Indirect effect | 10.8 | FOXP2 | intergenic | 16675 | NA | 7 | 114350502 |
| 10:8794106:C:T | Indirect effect | 10.79 | RP11-575N15.1 | intergenic | 27855 | NA | 10 | 8794106 |
| 15:47890350:G:T | Indirect effect | 10.79 | SEMA6D | intronic | 0 | NA | 15 | 47890350 |
| 15:78926726:C:G | Direct effect | 10.78 | CHRNB4 | intronic | 0 | NA | 15 | 78926726 |
| 2:146359296:A:C | Indirect effect | 10.78 | AC079163.1 | ncRNA_intronic | 0 | NA | 2 | 146359296 |
| 14:30148102:A:G | Indirect effect | 10.75 | PRKD1 | intronic | 0 | NA | 14 | 30148102 |
| 15:47796192:A:C | Indirect effect | 10.75 | SEMA6D | intronic | 0 | NA | 15 | 47796192 |
| 7:117800866:C:G | Indirect effect | 10.75 | NAA38 | intergenic | 23220 | NA | 7 | 117800866 |
| 11:28678870:A:G | Indirect effect | 10.74 | RP11-960D24.1 | intergenic | 21860 | NA | 11 | 28678870 |
| 10:8802912:G:T | Indirect effect | 10.72 | RP11-575N15.1 | intergenic | 36661 | NA | 10 | 8802912 |
| 1:44109749:A:G | Indirect effect | 10.72 | KDM4A | intergenic | 6080 | NA | 1 | 44109749 |
| 9:128723231:A:T | Indirect effect | 10.72 | PBX3 | intronic | 0 | NA | 9 | 128723231 |
| 18:35147621:C:G | Indirect effect | 10.71 | CELF4 | intergenic | 1621 | NA | 18 | 35147621 |
| 2:32341399:A:G | Indirect effect | 10.7 | SPAST | intronic | 0 | NA | 2 | 32341399 |
| 9:128656387:A:ATATT | Indirect effect | 10.68 | PBX3 | intronic | 0 | NA | 9 | 128656387 |
| 15:79019546:A:C | Direct effect | 10.67 | RP11-160C18.2 | ncRNA_exonic | 0 | NA | 15 | 79019546 |
| 15:79023415:C:T | Direct effect | 10.67 | RP11-160C18.2 | ncRNA_exonic | 0 | NA | 15 | 79023415 |
| 9:128366243:T:TACA | Indirect effect | 10.67 | MAPKAP1 | intronic | 0 | NA | 9 | 128366243 |
| 10:104456794:C:T | Indirect effect | 10.66 | ARL3 | intronic | 0 | NA | 10 | 104456794 |
| 7:118035388:A:C | Indirect effect | 10.66 | AC006926.1 | intergenic | 67165 | NA | 7 | 118035388 |
| 10:106495214:G:GTTTTTTT | Indirect effect | 10.65 | SORCS3 | intronic | 0 | NA | 10 | 106495214 |
| 2:203673072:A:C | Indirect effect | 10.65 | ICA1L | intronic | 0 | NA | 2 | 203673072 |
| 14:69609498:C:T | Indirect effect | 10.63 | DCAF5 | intronic | 0 | NA | 14 | 69609498 |
| 2:32355412:A:ATACT | Indirect effect | 10.62 | SPAST | intronic | 0 | NA | 2 | 32355412 |
| 9:128535858:A:C | Indirect effect | 10.62 | PBX3 | intronic | 0 | NA | 9 | 128535858 |
| 2:31943991:C:CT | Indirect effect | 10.59 | AL133247.3 | intergenic | 67060 | NA | 2 | 31943991 |
| 9:128445758:A:C | Indirect effect | 10.58 | MAPKAP1 | intronic | 0 | NA | 9 | 128445758 |
| 10:104670449:A:G | Indirect effect | 10.57 | RP11-724N1.1 | intergenic | 3893 | NA | 10 | 104670449 |
| 11:17094702:A:G | Indirect effect | 10.57 | AC116533.1 | upstream | 0 | NA | 11 | 17094702 |
| 2:145888369:C:T | Indirect effect | 10.57 | TEX41 | ncRNA_intronic | 0 | NA | 2 | 145888369 |
| 15:47558850:A:G | Indirect effect | 10.56 | SEMA6D | intronic | 0 | NA | 15 | 47558850 |
| 7:115109554:C:G | Indirect effect | 10.56 | RP11-222O23.1 | intergenic | 33867 | NA | 7 | 115109554 |
| 9:128288428:C:T | Indirect effect | 10.56 | MAPKAP1 | intronic | 0 | NA | 9 | 128288428 |
| 9:128629174:A:C | Indirect effect | 10.56 | PBX3 | intronic | 0 | NA | 9 | 128629174 |
| 11:112832813:C:T | Indirect effect | 10.55 | RP11-629G13.1 | ncRNA_intronic | 0 | NA | 11 | 112832813 |
| 2:203990789:G:T | Indirect effect | 10.55 | NBEAL1 | exonic | 0 | synonymous SNV | 2 | 203990789 |
| 4:35534331:A:T | Indirect effect | 10.55 | RNU6-573P | intergenic | 36706 | NA | 4 | 35534331 |
| 9:128723222:A:C | Indirect effect | 10.53 | PBX3 | intronic | 0 | NA | 9 | 128723222 |
| 9:127980889:G:GA | Indirect effect | 10.52 | RABEPK | intronic | 0 | NA | 9 | 127980889 |
| 10:104821946:A:G | Indirect effect | 10.5 | CNNM2 | intronic | 0 | NA | 10 | 104821946 |
| 15:78001011:C:T | Indirect effect | 10.5 | LINGO1 | intronic | 0 | NA | 15 | 78001011 |
| 18:50832102:A:G | Indirect effect | 10.5 | DCC | intronic | 0 | NA | 18 | 50832102 |
| 1:73851693:A:AATCT | Indirect effect | 10.49 | RP4-598G3.1 | intergenic | 30759 | NA | 1 | 73851693 |
| 11:112898428:A:T | Indirect effect | 10.48 | NCAM1 | intronic | 0 | NA | 11 | 112898428 |
| 15:79030049:G:T | Direct effect | 10.47 | RP11-160C18.2 | intergenic | 2212 | NA | 15 | 79030049 |
| 11:17297161:C:T | Indirect effect | 10.44 | NUCB2 | UTR5 | 0 | NA | 11 | 17297161 |
| 15:47576969:C:T | Indirect effect | 10.44 | SEMA6D | intronic | 0 | NA | 15 | 47576969 |
| 2:203995405:A:G | Indirect effect | 10.43 | NBEAL1 | intronic | 0 | NA | 2 | 203995405 |
| 7:117496319:C:T | Indirect effect | 10.43 | CTTNBP2 | intronic | 0 | NA | 7 | 117496319 |
| 8:43219175:T:TGAGTCA | Indirect effect | 10.43 | POTEA | downstream | 0 | NA | 8 | 43219175 |
| 17:18643813:A:C | Indirect effect | 10.42 | FBXW10 | intergenic | 3513 | NA | 17 | 18643813 |
| 10:104944720:A:G | Indirect effect | 10.41 | NT5C2 | intronic | 0 | NA | 10 | 104944720 |
| 1:73843213:G:T | Indirect effect | 10.41 | RP4-598G3.1 | intergenic | 22279 | NA | 1 | 73843213 |
| 3:48898749:C:T | Indirect effect | 10.41 | SLC25A20 | intronic | 0 | NA | 3 | 48898749 |
| 15:47677596:A:G | Indirect effect | 10.4 | CTD-2050N2.1 | ncRNA_intronic | 0 | NA | 15 | 47677596 |
| 2:32325414:A:G | Indirect effect | 10.4 | SPAST | intronic | 0 | NA | 2 | 32325414 |
| 3:50123417:A:G | Indirect effect | 10.4 | RBM6 | intronic | 0 | NA | 3 | 50123417 |
| 7:117979896:G:T | Indirect effect | 10.4 | ANKRD7 | intergenic | 97111 | NA | 7 | 117979896 |
| 18:50863861:A:G | Indirect effect | 10.37 | DCC | intronic | 0 | NA | 18 | 50863861 |
| 11:28643867:A:G | Indirect effect | 10.34 | RP11-960D24.1 | intergenic | 56863 | NA | 11 | 28643867 |
| 15:47684508:A:G | Indirect effect | 10.32 | CTD-2050N2.1 | ncRNA_intronic | 0 | NA | 15 | 47684508 |
| 1:96200401:A:T | Indirect effect | 10.32 | RP11-286B14.1 | ncRNA_intronic | 0 | NA | 1 | 96200401 |
| 3:49275828:A:G | Indirect effect | 10.32 | CCDC36 | intronic | 0 | NA | 3 | 49275828 |
| 9:128326190:C:T | Indirect effect | 10.32 | MAPKAP1 | intronic | 0 | NA | 9 | 128326190 |
| 3:49911449:C:G | Indirect effect | 10.31 | ACTBP13 | ncRNA_exonic | 0 | NA | 3 | 49911449 |
| 9:128024049:A:G | Indirect effect | 10.31 | GAPVD1 | upstream | 0 | NA | 9 | 128024049 |
| 7:117550244:A:G | Indirect effect | 10.3 | AC007568.1 | intergenic | 26515 | NA | 7 | 117550244 |
| 8:91943242:T:TATTTC | Indirect effect | 10.3 | NECAB1 | intronic | 0 | NA | 8 | 91943242 |
| 15:47873172:C:T | Indirect effect | 10.28 | SEMA6D | intronic | 0 | NA | 15 | 47873172 |
| 2:203709997:C:G | Indirect effect | 10.28 | ICA1L | intronic | 0 | NA | 2 | 203709997 |
| 15:79033018:A:G | Direct effect | 10.27 | RP11-160C18.2 | intergenic | 5181 | NA | 15 | 79033018 |
| 15:47488977:C:T | Indirect effect | 10.26 | SEMA6D | intronic | 0 | NA | 15 | 47488977 |
| 1:44072420:A:G | Indirect effect | 10.26 | PTPRF | intronic | 0 | NA | 1 | 44072420 |
| 10:104428716:C:T | Indirect effect | 10.25 | ARL3 | intergenic | 4772 | NA | 10 | 104428716 |
| 2:203673073:A:G | Indirect effect | 10.25 | ICA1L | intronic | 0 | NA | 2 | 203673073 |
| 5:166999828:C:T | Indirect effect | 10.25 | TENM2 | intronic | 0 | NA | 5 | 166999828 |
| 8:91857411:A:G | Indirect effect | 10.25 | RP11-662G23.1 | ncRNA_intronic | 0 | NA | 8 | 91857411 |
| 15:78760918:A:G | Direct effect | 10.24 | IREB2 | intronic | 0 | NA | 15 | 78760918 |
| 2:204015972:C:T | Indirect effect | 10.23 | NBEAL1 | intronic | 0 | NA | 2 | 204015972 |
| 4:147840979:A:T | Indirect effect | 10.23 | TTC29 | intronic | 0 | NA | 4 | 147840979 |
| 2:32347715:A:T | Indirect effect | 10.22 | SPAST | intronic | 0 | NA | 2 | 32347715 |
| 2:32432934:A:T | Indirect effect | 10.22 | SLC30A6 | intronic | 0 | NA | 2 | 32432934 |
| 7:117569397:A:T | Indirect effect | 10.22 | AC007568.1 | intergenic | 45668 | NA | 7 | 117569397 |
| 9:128675819:C:G | Indirect effect | 10.22 | PBX3 | intronic | 0 | NA | 9 | 128675819 |
| 7:114139684:A:G | Indirect effect | 10.2 | FOXP2 | UTR3 | 0 | NA | 7 | 114139684 |
| 5:154881240:A:G | Indirect effect | 10.18 | CTB-95D12.1 | intergenic | 8082 | NA | 5 | 154881240 |
| 7:114290415:C:T | Indirect effect | 10.18 | FOXP2 | intronic | 0 | NA | 7 | 114290415 |
| 14:69659659:A:G | Indirect effect | 10.17 | RP11-363J20.1 | ncRNA_intronic | 0 | NA | 14 | 69659659 |
| 2:203926271:A:G | Indirect effect | 10.17 | AC023271.1 | ncRNA_exonic | 0 | NA | 2 | 203926271 |
| 7:117512932:A:G | Indirect effect | 10.17 | CTTNBP2 | intronic | 0 | NA | 7 | 117512932 |
| 14:69629100:C:CAA | Indirect effect | 10.15 | DCAF5 | intergenic | 9257 | NA | 14 | 69629100 |
| 4:35529535:A:T | Indirect effect | 10.14 | RNU6-573P | intergenic | 31910 | NA | 4 | 35529535 |
| 5:154784177:A:G | Indirect effect | 10.14 | CTC-447K7.1 | intergenic | 6264 | NA | 5 | 154784177 |
| 15:47796491:G:T | Indirect effect | 10.13 | SEMA6D | intronic | 0 | NA | 15 | 47796491 |
| 2:22570074:A:G | Indirect effect | 10.13 | AC068490.2 | ncRNA_intronic | 0 | NA | 2 | 22570074 |
| 9:127962533:A:G | Indirect effect | 10.13 | RABEPK | upstream | 0 | NA | 9 | 127962533 |
| 3:49908023:A:G | Indirect effect | 10.12 | CAMKV | upstream | 0 | NA | 3 | 49908023 |
| 4:147943107:C:T | Indirect effect | 10.11 | RP11-292D4.3 | intergenic | 12810 | NA | 4 | 147943107 |
| 15:78894339:A:G | Direct effect | 10.1 | CHRNA3 | exonic | 0 | synonymous SNV | 15 | 78894339 |
| 9:128154808:A:G | Indirect effect | 10.1 | RP11-184B22.2 | intergenic | 19763 | NA | 9 | 128154808 |
| 15:79024355:A:G | Direct effect | 10.09 | RP11-160C18.2 | ncRNA_intronic | 0 | NA | 15 | 79024355 |
| 8:91919379:A:T | Indirect effect | 10.09 | NECAB1 | intronic | 0 | NA | 8 | 91919379 |
| 2:146375705:A:T | Indirect effect | 10.07 | AC079163.1 | intergenic | 14714 | NA | 2 | 146375705 |
| 9:128428404:A:G | Indirect effect | 10.07 | MAPKAP1 | intronic | 0 | NA | 9 | 128428404 |
| 15:91565804:A:G | Indirect effect | 10.06 | VPS33B | UTR5 | 0 | NA | 15 | 91565804 |
| 2:32579759:C:T | Indirect effect | 10.06 | BIRC6 | intergenic | 2337 | NA | 2 | 32579759 |
| 7:118090953:A:G | Indirect effect | 10.06 | AC006926.1 | intergenic | 11600 | NA | 7 | 118090953 |
| 8:43219230:G:T | Indirect effect | 10.05 | POTEA | downstream | 0 | NA | 8 | 43219230 |
| 7:117829908:C:T | Indirect effect | 10.04 | NAA38 | UTR3 | 0 | NA | 7 | 117829908 |
| 2:203662888:A:C | Indirect effect | 10.03 | ICA1L | intronic | 0 | NA | 2 | 203662888 |
| 9:127968109:C:T | Indirect effect | 10.03 | RABEPK | intronic | 0 | NA | 9 | 127968109 |
| 2:22439743:C:T | Indirect effect | 10.01 | AC068490.2 | ncRNA_intronic | 0 | NA | 2 | 22439743 |

Table S5. Prioritised Candidate Genes Identified for the Direct Component (Statistically Independent of Measured Current Smoking). Repeated symbols may represent distinct tissue/transcript prediction models; MAGMA/FOCUS prioritisation does not establish causality.

| **Gene Symbol** | **Chromosome** | **Genomic Position** | **CV R²** | **CV R² P-value** | **TWAS Z-score** | **PIP** | **SNPs (n)** | **Parameters (n)** | **MAGMA Z-statistic** | **MAGMA P-value** | **MAGMA FDR** |
| --- | --- | --- | --- | --- | --- | --- | --- | --- | --- | --- | --- |
| IREB2 | 15 | chr15:78729773-78793798 | 0.132 | 3.47e-11 | -7.22 | 1 | 111 | 10 | 6.11 | 5.00e-10 | 1.14e-06 |
| CLPTM1L | 5 | chr5:1317859-1345214 | 0.219 | 2.99e-18 | -6.15 | 1 | 58 | 7 | 6.34 | 1.11e-10 | 6.79e-07 |
| MORF4L1 | 15 | chr15:79102829-79190475 | 0.063 | 5.36e-06 | -5.75 | 0.952 | 302 | 13 | 5.42 | 2.98e-08 | 6.06e-05 |
| HYKK | 15 | chr15:78799906-78829714 | 0.048 | 6.24e-05 | -3.56 | 1 | 66 | 6 | 7.67 | 8.60e-15 | 1.58e-10 |
| PSMA4 | 15 | chr15:78832747-78841604 | 0.161 | 1.94e-13 | 3.38 | 1 | 24 | 3 | 6.69 | 1.14e-11 | 1.05e-07 |
| PSMA4 | 15 | chr15:78832747-78841604 | 0.498 | 1.73e-47 | -2.61 | 1 | 24 | 3 | 6.69 | 1.14e-11 | 1.05e-07 |
| ADAMTS7 | 15 | chr15:79051545-79103773 | 0.092 | 3.95e-08 | -2.07 | 0.771 | 126 | 9 | 6.11 | 5.00e-10 | 1.14e-06 |

Table S6. Prioritised Candidate Genes Identified for the Indirect Component (Associated with Measured Current Smoking). Repeated symbols may represent distinct tissue/transcript prediction models; MAGMA/FOCUS prioritisation does not establish causality.

| **Gene Symbol** | **Chromosome** | **Genomic Position** | **CV R²** | **CV R² P-value** | **TWAS Z-score** | **PIP** | **SNPs (n)** | **Parameters (n)** | **MAGMA Z-statistic** | **MAGMA P-value** | **MAGMA FDR** |
| --- | --- | --- | --- | --- | --- | --- | --- | --- | --- | --- | --- |
| HSPA5 | 9 | chr9:127997132-128003609 | 0.05 | 4.58e-05 | 6.9 | 0.87 | 5 | 2 | 4.9 | 4.89e-07 | 1.09e-04 |
| TRIM8 | 10 | chr10:104404253-104418164 | 0.068 | 2.19e-06 | 6.88 | 0.964 | 18 | 6 | 4.82 | 7.13e-07 | 1.42e-04 |
| NT5C2 | 10 | chr10:104845940-104953056 | 0.224 | 1.24e-18 | -6.72 | 0.999 | 222 | 17 | 5.25 | 7.68e-08 | 2.81e-05 |
| PPP6C | 9 | chr9:127908852-127952218 | 0.31 | 1.72e-26 | 6.01 | 0.966 | 73 | 6 | 6.55 | 2.86e-11 | 6.55e-08 |
| PPP1R13B | 14 | chr14:104200089-104313927 | 0.341 | 1.71e-29 | 5.83 | 0.993 | 201 | 9 | 4.64 | 1.77e-06 | 2.82e-04 |
| PBX3 | 9 | chr9:128509624-128729656 | 0.222 | 1.76e-18 | -5.81 | 0.901 | 220 | 16 | 5.97 | 1.22e-09 | 8.93e-07 |
| PPP1R13B | 14 | chr14:104200089-104313927 | 0.374 | 7.51e-33 | 5.74 | 0.992 | 201 | 9 | 4.64 | 1.77e-06 | 2.82e-04 |
| PPP1R13B | 14 | chr14:104200089-104313927 | 0.056 | 1.85e-05 | -5.67 | 0.998 | 201 | 9 | 4.64 | 1.77e-06 | 2.82e-04 |
| AS3MT | 10 | chr10:104629273-104661656 | 0.849 | 8.01e-127 | 5.43 | 0.731 | 94 | 11 | 5.47 | 2.28e-08 | 1.13e-05 |
| ARID5B | 10 | chr10:63661059-63856703 | 0.013 | 2.38e-02 | 5.02 | 1 | 428 | 47 | 3.56 | 1.84e-04 | 8.49e-03 |
| PIK3C2A | 11 | chr11:17099277-17229530 | 0.013 | 2.41e-02 | -4.7 | 0.977 | 187 | 15 | 4.86 | 5.95e-07 | 1.23e-04 |
| USP34 | 2 | chr2:61414598-61697904 | 0.167 | 6.35e-14 | -4.44 | 0.957 | 520 | 21 | 3.06 | 1.09e-03 | 2.94e-02 |
| USP34 | 2 | chr2:61414598-61697904 | 0.168 | 5.02e-14 | -4.43 | 0.971 | 520 | 21 | 3.06 | 1.09e-03 | 2.94e-02 |
| USP34 | 2 | chr2:61414598-61697904 | 0.129 | 6.60e-11 | 4.36 | 0.965 | 520 | 21 | 3.06 | 1.09e-03 | 2.94e-02 |
| MDFIC | 7 | chr7:114562209-114659256 | 0.182 | 3.53e-15 | -4.35 | 0.984 | 130 | 24 | 4.44 | 4.48e-06 | 5.81e-04 |
| MDFIC | 7 | chr7:114562209-114659256 | 0.261 | 6.32e-22 | 3.96 | 0.93 | 130 | 24 | 4.44 | 4.48e-06 | 5.81e-04 |
| TRAF3IP2 | 6 | chr6:111877657-111927481 | 0.058 | 1.34e-05 | -3.77 | 0.801 | 126 | 16 | 3.88 | 5.29e-05 | 3.40e-03 |
| CUEDC2 | 10 | chr10:104183002-104192418 | 0.089 | 6.20e-08 | 3.68 | 0.914 | 13 | 4 | 3.03 | 1.22e-03 | 3.12e-02 |
| ADO | 10 | chr10:64564516-64568238 | 0.391 | 1.03e-34 | 3.48 | 0.888 | 7 | 2 | 3 | 1.36e-03 | 3.33e-02 |
| VRK2 | 2 | chr2:58134786-58387055 | 0.117 | 5.48e-10 | 3.42 | 0.899 | 445 | 27 | 3.91 | 4.66e-05 | 3.15e-03 |
| TGFBRAP1 | 2 | chr2:105880871-105946491 | 0.161 | 2.00e-13 | 2.37 | 0.886 | 180 | 22 | 3.67 | 1.22e-04 | 6.37e-03 |
| C2orf49 | 2 | chr2:105953816-105965668 | 0.119 | 3.34e-10 | -2.02 | 0.841 | 29 | 8 | 3.73 | 9.49e-05 | 5.33e-03 |

**Figure S1.** Functional Annotation and Predicted Deleteriousness of SNPs Associated with the Direct and Indirect Components. **(A)** The distribution of Combined Annotation Dependent Depletion (CADD) scores for all component-specific SNPs. The histogram shows that variants from the indirect component (blue) have a distribution skewed towards higher CADD scores compared to the direct component (red), indicating a greater proportion of variants with high predicted deleteriousness. **(B)** Functional annotation of SNPs, revealing that the majority of variants for both components are located in non-coding (intronic and intergenic) regions. **(C)** The top SNPs from the indirect component ranked by CADD score, highlighting variants predicted to be highly deleterious (CADD > 20). **(D)** Chromosomal distribution of SNPs, stacked by CADD score category. The plot highlights the strong concentration of direct-component SNPs on chromosome 15, in contrast to the more polygenic distribution of indirect-component SNPs across multiple chromosomes.

**Figure S2.** Tissue-Specific Gene Expression Enrichment for the Direct and Indirect Genetic Components. The y-axis represents the -log10 transformed Bonferroni-corrected P-value, with the dashed line indicating the significance threshold. **(A)** Genes associated with the direct component (statistically independent of measured current smoking) display a pattern of dual enrichment, with the most significant signal in Lung tissue, followed by significant enrichment in the Brain Cerebellar Hemisphere and Cerebellum. **(B)** Genes associated with the indirect component (associated with measured current smoking) show profound and specific enrichment broadly across numerous brain tissues.
